# Identifying pathogenic processes by integrating microarray data with prior knowledge

**DOI:** 10.1186/1471-2105-15-115

**Published:** 2014-04-24

**Authors:** Ståle Nygård, Trond Reitan, Trevor Clancy, Vegard Nygaard, Johannes Bjørnstad, Biljana Skrbic, Theis Tønnessen, Geir Christensen, Eivind Hovig

**Affiliations:** 1 Bioinformatics Core Facility, Institute for Medical Informatics, Oslo University Hospital, Oslo, Norway; 2 Institute for Experimental Medical Research, Oslo University Hospital and University of Oslo, Oslo, Norway; 3 KG Jebsen Cardiac Research Centre and Center for Heart Failure Research, University of Oslo, Oslo, Norway; 4 Center for Ecological and Evolutionary Synthesis, Department of Biology, University of Oslo, Oslo, Norway; 5 Department of Tumor Biology, Institute for Cancer Research, Oslo University Hospital, Oslo, Norway; 6 Department of Cardiothoracic Surgery, Oslo University Hospital, Oslo, Norway; 7 Institute for Medical Informatics, Oslo University Hospital, Oslo, Norway; 8 Department of informatics, University of Oslo, Oslo, Norway

## Abstract

**Background:**

It is of great importance to identify molecular processes and pathways that are involved in disease etiology. Although there has been an extensive use of various high-throughput methods for this task, pathogenic pathways are still not completely understood. Often the set of genes or proteins identified as altered in genome-wide screens show a poor overlap with canonical disease pathways. These findings are difficult to interpret, yet crucial in order to improve the understanding of the molecular processes underlying the disease progression. We present a novel method for identifying groups of connected molecules from a set of differentially expressed genes. These groups represent functional modules sharing common cellular function and involve signaling and regulatory events. Specifically, our method makes use of Bayesian statistics to identify groups of co-regulated genes based on the microarray data, where external information about molecular interactions and connections are used as priors in the group assignments. Markov chain Monte Carlo sampling is used to search for the most reliable grouping.

**Results:**

Simulation results showed that the method improved the ability of identifying correct groups compared to traditional clustering, especially for small sample sizes. Applied to a microarray heart failure dataset the method found one large cluster with several genes important for the structure of the extracellular matrix and a smaller group with many genes involved in carbohydrate metabolism. The method was also applied to a microarray dataset on melanoma cancer patients with or without metastasis, where the main cluster was dominated by genes related to keratinocyte differentiation.

**Conclusion:**

Our method found clusters overlapping with known pathogenic processes, but also pointed to new connections extending beyond the classical pathways.

## Background

High-throughput experimental techniques provide objective views of the molecular changes that occur in cells during disease progression. A widely used tool when trying to understand the biological meaning of the observed changes is pathway analysis. In pathway analysis, the list of significantly altered genes or molecules is mapped onto usually pre-compiled pathways. A wide range of methods for finding pre-defined pathways overrepresented by significant genes have been developed and are steadily used, e.g. [1-3]. However, this strategy for identifying the important disease pathways is limited, as pathways are not fixed and change with context [4]. As a consequence, many of the significantly altered genes or molecules fall outside of the expected pathways [5].

An alternative approach, which is fundamentally different from the pathway analysis just described, is a purely data-driven approach, in which the experimental data is used without any guidance from prior knowledge about molecular interactions. The goal is then to discover hidden patterns of correlation between genes reflecting the complex processes and pathways that underlie cellular metabolism and physiology [6]. The most common approach for this task is unsupervised clustering. This class of methods was of the first to be applied to microarray data [7], and several approaches have been proposed, e.g hierarchical and *k*-means clustering, Prediction Around Medoids (PAM) [8], model-based clustering via Markov chain Monte Carlo (MCMC), e.g [9,10], tight clustering [11] and clustering via networks [12]. Another way of learning molecular relationships from microarray data is to use Bayesian network (BN) methodology. Instead of finding groups of correlated molecules, BN methodology infers a network of (direct) interacting molecules. BNs were first proposed for microarray data in 2000 [13], and have also been much applied.

Along with the development of such unsupervised methods, there has been a steady development of prior information about molecular interactions. In addition to the mentioned pathways, there are many databases containing pairwise connections, such as protein-protein interactions, transcription factors binding to genes and protein sequence similarities. Such information is maybe a more reliable source of information than pathways, as the latter lack proper definitions, and are as already mentioned heavily context dependent.

Bayes’ formula is well suited for taking prior information into account during inference, and says

$$p\;\left(M,|,D\right)=\frac{p\;\left(D,|,M\right)p\left(M\right)}{p\;\left(D\right)}\propto p\;\left(D,|,M\right)p\;\left(M\right).$$

The goal of the analysis is the posterior probability *p*(*M*|*D*), i.e. the probability that the model (*M*) is correct given the data (*D*). In our setting, the model *M* represents a clustering or a network configuration, whereas *D* represents the microarray data. Many methods using BN methodology to incorporate informative priors have been proposed quite recently, e.g [14-17]. However, even with the help of prior information, inferring Bayesian networks for large numbers of genes remains computationally challenging, and the number of genes that can be included is usually smaller than one hundred. Inference about groups or clusters of genes is a less daunting task, as there are fewer group/cluster configurations than network configurations. For cluster analysis, there has been some, but not many, suggestions for how to make use of prior knowledge, e.g [18]. Many Bayesian model-based clustering methods have been proposed, e.g. [9,10,19,20] but common to all of these is that they use non-informative priors. Bayesian clustering with truly informative priors has to our knowledge not previously been proposed.

In the present paper, we develop a novel method for clustering using Bayesian statistics and informative priors making use of Markov chain Monte Carlo (MCMC) sampling. We name our method MCIP (MCMC Clustering with Informative Priors). In our method, co-regulation patterns in the microarray data are used together with prior knowledge to find groups or modules of interacting genes. Specifically, we propose a method which searches for an optimal partitioning of the genes into functional modules, where module assignment is based on both microarray data and prior knowledge. The prior value for assigning genes to the same module is retrieved from databases containing gene pairs with a previously reported interaction or connection. Markov chain Monte Carlo (MCMC) sampling is used to search for the most reliable modules. Having found the modules, we generate subnetworks consisting of the prior pairs within each module, hypothesizing that these pairs represent the direct interactions. In the presented work we use prior information in forms of protein-protein interaction data, transcription factor binding predictions and protein sequence similarities. We apply our method to simulated data as well as two real-world microarray data sets, one in heart failure and one in melanoma cancer.

## Methods

Assume we have a genomic dataset *D*, e.g. a microarray dataset, with measurements of *n* genes. We want to use *D* together with prior knowledge about molecular interactions to find the most likely partitioning into groups or modules of functionally related genes. Now assume that the *n* genes represent *k* different modules, where *K* is not known in advance. Let *M*={(*i*_*m*_,*j*_*m*_,*p*_*m*_),*m*∈{1,…,*q*}} be the collection of *q* gene pairs for which prior knowledge exist, where $p_{m}=p_{i_{m},j_{m}}$ is the prior probability that gene *i*_*m*_ and gene *j*_*m*_ belong to the same module.

Now let ***g*** =(*g*_1_,…,*g*_*n*_) be a representation of group/module membership for each of the *n* genes, where *g*_*j*_∈{1,…,*K*}. We want to find the most likely division ***g*** into modules given the data as well as the prior knowledge, or formally, find the ***g*** maximizing *P*(***g*** ∣*D*,*M*). Using Bayes’ formula, we have

$$P\;\left(g,|,D,,,M\right)=\frac{P\;\left(D,|,g\right)\;P\;\left(g,|,M\right)}{P\;\left(D,|,M\right)}\propto P\;\left(D,|,g\right)P\;\left(g,|,M\right).$$

### Marginal likelihood given grouping

We start by finding the marginal likelihood given the subdivision into different groups (modules/clusters) and the prior information, i.e. *P*(*D*∣ ***g***,*M*). Assume the data in group *k* follow a multivariate normal distribution with covariance matrix *Σ*_*k*_, whose prior is inverse Wishart *f*(*Ψ*_*k*_,*m*_*k*_) where *Ψ*_*k*_ describes the prior covariance matrix and *m*_*k*_ describes the sharpness of the prior. Assume also that all prior information about the data is contained in *M*, and that genes belonging to different groups are independent of each other. Consequently, we can let each group *k* have prior covariance matrix *Ψ*_*k*_=*ρ*_*k*_*I*_*k*_, where *I*_*k*_ is a *n*_*k*_×*n*_*k*_ identity matrix and *n*_*k*_ is the number of genes in group *k*. We thus have that

(1) $$\;P\;\left(D,|,g\right)=\overset{K}{\underset{k=1}{\prod }}\frac{|\Psi_{k}{|}^{m_{k}/2}\Gamma_{n_{k}}\left(\left(m_{k},+,N\right),/,2\right)}{\pi^{\left(N,\cdot ,n_{k}\right)/2}|\Psi_{k}|+X_{k}^{t}X_{k}^{\left(m_{k},+,N\right)/2}\Gamma_{n_{k}}\left(m,/,2\right)},$$

where *N* is the number of rows in *D* (number of individuals), *X*_*k*_ is the data matrix for genes belonging to group *k* and *Γ*_*i*_() is the multivariate gamma function. If the gene expressions are standardized to have mean values equal to zero and variances equal to one, it is natural to choose *ρ*_*k*_=1 and *m*_*k*_=*n*_*k*_+2, as this yields expected values of 1 for the variances, i.e. for the diagonal elements. The expectancy of the inverse Wishart distribution is defined only for *m*_*k*_>*n*_*k*_+1, so this is a minimalistic way of coding for the knowledge that the data has been standardized.

### Probability of grouping given prior information

Next, we want to find *P*(***g*** ∣*M*), i.e. the probability of grouping ***g*** given the prior information *M*. Assume we have *n* genes and *K*∈{1,…,*n*} groups. Let *N*(*n*,*K*) denote the number of possible subdivisions of *n* genes into *K* groups. A new gene can be inserted into one of the *K* existing groups, or as its own, single-membered, group, so *N*(*n*,*K*) can be defined recursively by

$$N\;\left(n,,,K\right)=K\cdot N\;\left(n,-,1,,,K\right)+N\;\left(n,-,1,,,K,-,1\right),$$

where *N*(*K*,*K*)=1. Let *N*_*i* *j*_(*n*,*K*)=*K*·*N*(*n*-2,*K*)+*N*(*n*-2,*K*-1)=*N*(*n*-1,*K*) be the number of subdivisions of *n* genes into *K* groups where *i* and *j* are in the same group. Let $N\;\left(n\right)=\sum_{K=1}^{n}N\;\left(n,,,K\right)$ be the number of subdivisions of *n* genes in total and $N_{\text{ij}}\left(n\right)=\sum_{K=1}^{n}N_{\text{ij}}\left(n,,,K\right)=\sum_{K=1}^{n}N\;\left(n,-,1,,,K\right)=N\;\left(n,-,1\right)$ be the number of subdivisions of *n* genes where *i* and *j* are in the same group.

Now let *M*={(*i*_*m*_,*j*_*m*_,*p*_*m*_),*m*∈{1,…,*q*}} be the set of *q* pairs for which prior knowledge exist, where we define *p*_*m*_ as the prior probability of forcing gene *i*_*m*_ and *j*_*m*_ to belong to the same group. Consider first the situation where we have no prior knowledge, i.e. *M*_0_=*∅*. Let (*K*,*n*) denote that we have *K* groups of *n* objects, and let *P*(*K*∣*M*_0_)=1/*n*, i.e equal prior probability for the number of groups. For a particular grouping ***g*** =(*g*_1_,…,*g*_*n*_) that has a total of *K*_*g*_ groups, let *P*(***g*** ∣*M*_0_,*K*_*g*_))=*I*(*K*=*K*_*g*_)/*N*(*n*,*K*_*g*_), i.e. equal probability for all groupings for a given number of groups *K*_*g*_. This implies that the probability of grouping ***g*** given no prior knowledge is

$$P\;\left(g(|(M_{0}\right)=\frac{1}{n\cdot N\;\left(n,,,K_{g}\right)}.$$

We denote this probably measure $P_{M_{0}}$ and refer to it as the baseline prior.

We will now specify a probability measure which has the same structure as the baseline prior, except that *i* and *j* are forced to be in the same group and will thus be handled as a unit. To do this, consider the situation where we have one pair of genes that belong to the same group with probability one. The prior knowledge is then defined as *M*_1,1_={(*i*,*j*),*p*_1_=1}. We then have in total *N*_*i* *j*_(*n*)=*N*(*n*-1) possible subdivisions and *n*-1 possible number of groups. The number of subdivisions into *K* groups is *N*_*i* *j*_(*n*,*K*)=*N*(*n*-1,*K*). This means that

$$P\;\left(K,|,M_{1,1}\right)=\frac{1}{n-1}I\;\left(K,\leq ,n,-,1\right),$$

$$\;P\;\left(g,\;,|,\;,M_{1,1},,,K\right)\;=\;\frac{1}{N_{\text{ij}}\left(n,,,K\right)}I\;\left(g_{i},\;,=,\;,g_{j}\right)\;=\;\frac{1}{N\;\left(n,-,1,,,K\right)}I\;\left(g_{i},\;,=,\;,g_{j}\right),$$

where *I* is the indicator function, so

$$\;P\;\left(g,|,M_{1,1}\right)=\frac{1}{\left(n,-,1\right)N\;\left(n,-,1,,,K_{g}\right)}I\;\left(K_{g},\leq ,n,-,1,,,g_{i},=,g_{j}\right).$$

Call this probability measure $P_{M_{1,1}}$.

Then consider the situation where we have one pair of genes that belong to the same group with probability *p*_1_≠1. We now define the prior knowledge as *M*_1_={(*i*,*j*),*p*_1_(≠1)}. The idea is that $P_{M_{1}}=p_{1}P_{M_{1,1}}+\;\left(1,-,p_{1}\right)P_{M_{0}}$, that is a mixture between a probability measure which forces *i* and *j* to be in the same group and a probability measure that treats all genes equally (the baseline prior). Following this idea, we have

$$\;P\;\left(g,\;,|,\;,M_{1}\right)\;=\;\frac{p_{1}I\;\left(K_{g},\leq ,n,-,1,,,g_{i},=,g_{j}\right)}{\left(n,-,1\right)N\;\left(n,-,1,,,K_{g}\right)}+\left(1,-,p_{1}\right)\frac{1}{n\cdot N\;\left(n,,,K_{g}\right)}.$$

Now let’s generalize to the situation where we have *q* pairs of genes with existing prior knowledge, i.e. we have *M*={(*i*_*m*_,*j*_*m*_,*p*_*m*_),*m*∈{1,…,*q*}} with $p_{m}=p_{i_{m},j_{m}}$. Since we have a probability for each pair, we need to introduce some notation specifying what pairs in the prior that are forced to be in the same group. This can be achieved by introducing *X*={*X*_*m*_},*m*∈1,…,*q*, where *X*_*m*_∈{0,1} indicates whether the pair (*i*_*m*_,*j*_*m*_) is forced to be in the same group or not, and *M*_*X*_={(*i*_*m*_,*j*_*m*_,*p*_*m*_)|*X*_*m*_=1} are the pairs that are forced together. We also define the total number of forced pairs for such a combination *X*as $x=\sum_{m=1}^{q}X_{m}.$ We have that

$$P\;\left(K,,,|,M_{x}\right)=\frac{1}{n-x}I\;\left(K,\leq ,n,-,x\right),$$

$$P\;\left(g,|,M_{X},,,K\right)=\frac{I\;\left(K_{g},<,n,-,x,,,g_{i_{m}},=,g_{j_{m}},,\forall ,,m,|,X_{m},=,1\right)}{N\;\left(n,-,x,,,K_{g}\right)},$$

and thus

$$P\;\left(g,|,M_{X}\right)=\frac{I\;\left(K_{g},<,n,-,x,,,g_{i_{m}},=,g_{j_{m}},,\forall ,,m,|,X_{m},=,1\right)}{\left(n,-,x\right)N\;\left(n,-,x,,,K_{g}\right)}.$$

Since we have

$$P\;\left(M_{X}\right)=\overset{q}{\underset{m=1}{\prod }}p_{m}^{X_{m}}{\left(1,-,p_{m}\right)}^{1-X_{m}},$$

and $P\;\left(g,|,M\right)=\underset{X}{\sum }P\;\left(g,|,M_{X}\right)P\;\left(M_{X}\right)$, we arrive at

(2) $$\begin{array}{c} P\left(g,|,M\right)=\underset{X}{\sum }\;\left(\overset{q}{\underset{m=1}{\prod }}p_{m}^{X_{m}}{\left(1,-,p_{m}\right)}^{1-X_{m}}\right)\\ \;\times \frac{I\;\left(K_{g},\leq ,n,-,x,,,g_{i_{m}},=,g_{j_{m}},,\forall ,,m,|,X_{m},=,1\right)}{\left(n,-,x\right)N\;\left(n,-,x,,,K_{g}\right)}. \end{array}$$

Note that this expression increases exponentially with the number of prior pairs *q*. In order to avoid computational cost increasing exponentially with the number of prior pairs, we developed a Monte Carlo estimation of *P*(***g*** ∣*M*), described in Additional file 1.

For a given grouping, the baseline prior contributes to the overall probability of a grouping with an additive factor (1-*p*_1_)(1-*p*_2_)⋯(1-*p*_*q*_)*P*(*g*|*M*_0_) (see Eq. 2 and the expression for the baseline prior *P*(*g*|*M*_0_)). This implies that the baseline prior will contribute to the probability of two genes being clustered. Define *P*_*b*_ as the probability for two genes being in the same group given the baseline prior *M*_0_. It is important that this probability is non-zero, in order to allow for the possibility of two arbitrary genes being in the same group whether they are in the set of prior pairs or not. If not, it would be impossible to group gene pairs that are not in the set of specified prior pairs. However, this implies that the probability of grouping two genes (*i*_*m*_, *j*_*m*_) in a prior pair will be be the mixture *p*_*m*_+(1-*p*_*m*_)*P*_*b*_, as there is a non-zero probability that the genes are connected, even if they should not be connected according to the prior information. For instance, if the baseline prior for two arbitrary genes to be connected is 0.1 and pair number *m* has prior enforcement probability *p*_*m*_=0.8, the total prior probability for the pair (*i*_*m*_,*j*_*m*_) to be connected will be 0.82.

There will be a baseline probability for two arbitrary genes to be connected. We have become aware that with equal probability for all number of groups and equal probability for each grouping given the number of groups, the baseline for any two genes to be connected will be dependent on the total number of genes, *n*. In general, we get that the prior for groupings gives that the baseline probability, *P*_*b*_ for two genes to be connected when there are *K* groups will be P(*i* and *j* in the same group ∣*K* groups)= *N*(*n*-1,*K*)/*N*(*n*,*K*). The total probability will then be $P_{b}=\left(1,/,n\right)\sum_{K}N\;\left(n,-,1,,,K\right)/N\;\left(n,,,K\right)$, which depends on the number of genes in total, *n*. For *n*=100, *P*_*b*_=0.048 while for the extreme case *n*=10, *P*_*b*_=0.25. It might be seen as a weakness that there is any dependency on the number of genes in the dataset on the baseline probability. An alternative approach could be to specify the baseline prior according to a given baseline probability for any arbitrary pair of genes. This would make for a more consistent baseline prior, but has the disadvantage that the baseline prior has to be set manually. An alternative would be to make the baseline prior into a parameter to be estimated in a hierarchical Bayesian model.

### Removing cycles

The set of priors might contain cycles, which can easily occur, e.g if both direct and indirect connections are included. We have developed an algorithm for detecting cycles, and if such are found, the prior pair with the smallest prior probability (smallest extra connection probability) is removed, as this connection is interpreted as a result of the rest of the cycle. The reason for excluding cycles in the prior is that with cycles, the pairwise specification of prior probabilities of pairs could be misleading. As an example, let us assume the prior is specified so that there is a 0.8 prior probability of a pairing between gene A and B (prior pair 1), between B and C (prior pair 2) and between A and C (prior pair 3). Then the probability for a forced connection between A and B (thus disregarding the baseline probability) will be P(A and B forced to be in the same group) = P(prior pair 1 enforced) + P(prior pair 1 not enforced)P(prior pair 2 enforced)P(prior pair 3 enforced) = 0.8 + 0.2*(0.8*0.8) = 0.928. Thus in order for the pairwise prior probabilities to be interpreted as the probability for two pairs to be forced to be connected, cycles should be avoided.

### Markov chain Monte Carlo procedure for integrative networks

We use Markov chain Monte Carlo (MCMC) to sample from the posterior distribution *P*(*D*∣ ***g***,*M*), as the analytical solution is not known. We will use the Metropolis Hastings algorithm, which is the most general version of MCMC. Specifically, we propose the following algorithm: 

• Start with a random grouping, called ***g***.

• Sample *N* groupings ***g*** based on the following scheme: 

Propose either (each with probability 1/3) 

∗ A partitioning of one of the groups (except when *K*=*n*)

∗ A merging of two of the groups (except when *K*=1)

∗ A transition of one of the genes to another group (except when *K*=1)

– Call the new grouping ***g***_new_.

– Accept ***g***_new_ with the following probability:

$$\;P\;\left(\text{accept},g_{\text{new}}\right)\;=\;\min\;\left(\;1,\frac{P\;\left(D,\;,|,\;,g_{\text{new}}\right)P\;\left(g_{\text{new}},|,M\right)Q\;\left(g_{\text{old}},\;,|,\;,g_{\text{new}}\right)}{P\;\left(D,\;,|,\;,g_{\text{old}}\right)P\;\left(g_{\text{old}},\;,|,\;,M\right)Q\;\left(g_{\text{new}},\;,|,\;,g_{\text{old}}\right)}\;\right)\;,$$

where *P*(*D*∣ ***g***) is given by (1), *P*(***g*** ∣*M*) is given by (2) and *Q*(***g***_x_| ***g***_y_) is the probability of proposing the group configuration ***g***_x_ from the configuration ***g***_y_, and is given by (3-5) in the next section.

In order to avoid convergence to local maxima, we implemented parallel tempering [21], as described in Additional file 1.

### Inferring clusters from MCMC samples

The above MCMC procedure results in a series of samples ***g***. These samples can be used to calculate the posterior similarity matrix (PSM), in which each entry (*i*,*j*) gives the proportion *p*_*i* *j*_ of samples gene *i* and gene *j* occur together in the same cluster. We infer clusters from the PSM using the *minbinder* function in the mcclust R library [22]. Specifically, *minbinder* makes use of hierarchical clustering with the PSM as distance matrix together with the *cuttree* function to produce cluster configurations with the number of clusters ranging from 1 to *L*, where *L* is a user-specified maximum. Now letting *I*_*K*_(*i*,*j*) be the indicator for whether gene *i* and *j* are in the same cluster for the configuration using *K* clusters (based on hierarchical clustering and the *cuttree* function), and using absolute difference as loss function, the posterior expected loss *e*_*K*_ for *K* clusters is calculated in *minbinder* as $e_{K}=\sum_{i<j}|I_{K}\left(i,,,j\right)-p_{\text{ij}}|$. The inferred cluster configuration is the result of hierarchical clustering and *cuttree* for $\hat{K}$ clusters, where $\hat{K}$ is the *k* minimizing the posterior expected loss, i.e. $\hat{K}=\underset{K}{\min}e_{K}$.

### Proposal distribution

Let *n*, as before, be the total number of genes, and let *n*_*k*_ be the number of genes in group *k* and *n*_*s*_ be the number of single-membered groups. If the number of groups *K* equals 1, the only allowed option is splitting into two new groups. If *K*=*n*, we can only have a merging of two genes into a new group. If 1<*K*<*n*, all three types of moves are allowed (splitting and merging groups, and moving a gene into another group). Each type of move then has probability 1/3.

First, consider the situation where ***g*** → ***g***^′^ is due to a splitting of group *k*. For the configuration ***g***, we then either have 1<*K*<*n*, and a splitting is occurring with probability 1/3, or *K*=1, and this type of move is occurring with probability 1. The probability of getting the new grouping ***g***^′^ is then the product of the probability of having a split within group *k*, which is *n*_*k*_/(*n*-*n*_*s*_), the probability of the actual splitting of j into two specific new subgroups, which is 1/*N*(*n*_*k*_,2), and the probability of having a split (rather than a merge or a transition of one of the genes), which is 1/3·*I*(1<*K*<*n*)+*I*(*K*=1). That is, we have,

(3) $$\begin{array}{c} Q\;\left(g,|,g^{\prime }\right)=\frac{n_{k}}{n-n_{s}}\cdot \frac{1}{N\;\left(n_{k},,,2\right)}\left(0,\cdot ,I,\;,\left(K,=,n\right)\right)\\ \;\left(+,\frac{1}{3},I,\;,\left(1,<,K,<,n\right),+,I,\;,\left(K,=,1\right)\right). \end{array}$$

Next, consider the situation where ***g*** → ***g***^′^ is due to a merging of group *k* and *l*. A merging happens with probability one if the number of groups is equal to n and with probability 1/3 if 1<*k*<*n*. Two groups, *l* and *k*, are chosen by first sampling a random gene and finding which group the gene belongs to, then picking another random gene and re-sampling as long as that other gene belongs to the same group. The probability of merging group *l* and group *j* has thus a proposal probability P(merge *l* and *j*) = P(choose *l* and *k* ∣ merge)P(merge) = [P(choose *k* first)P(choose *l* second ∣*k* chosen first) + P(choose *l* first)P(choose *k* second ∣*l* chosen first)] P(merge). That is, we have

(4) $$\begin{array}{c} Q\;\left(g,|,g^{\prime }\right)=\left(\frac{n_{k}}{n}\cdot \frac{n_{l}}{n-n_{k}}+\frac{n_{l}}{n}\cdot \frac{n_{k}}{n-n_{l}}\right)\\ \;\times \;\left(I\left(K,=,1\right)+\frac{1}{3}I\;\left(1,<,K,<,n\right)\right) \end{array}$$

Finally, consider the situation where ***g*** → ***g***^′^ is the result of moving a gene in group *l* to group *k*. A move of one gene from group *l* to group *k* is proposed by sampling a random gene and re-sampling if the gene itself constitutes a single-membered group. Another random gene not belonging to group *l* is then sampled, defining another group *k*. A random element from group *l* is then assigned group identity *k*. The proposal probability thus becomes P(move the *k*th gene of group *l* to group *k*) = P(choose group *l* to move from and group *k* to move to and choose *i*th element of group *l* ∣ move)P(move) = P(choose group *l*)P(choose group *k* ∣ group *l*)P(choose *i*th element from group *l*)P(move) = (*n*_*l*_/(*n*-*n*_*s*_))(*n*_*k*_/(*n*-*n*_*l*_))(1/*n*_*l*_)(1/3)*I*(1<*K*<*n*). Thus, we have

(5) $$Q\;\left(g,|,g^{\prime }\right)=\frac{n_{k}}{\left(n,-,n_{s}\right)\;\left(n,-,n_{l}\right)}\frac{1}{3}I\;\left(0,<,K,<,n\right).$$

## Computational aspects

The likelihood estimation involves the determinant function, which is *O*(*n*^3^) in the number *n* of genes in each module. In our method, the most computationally expensive calculation is the exact calculation of the prior probability *P*(***g*** |*M*). This calculation is exponential in the number of prior *pairs*. Often, as is the case for the data used in this paper, the number of pairs for which there exist prior knowledge, can be substantial. To deal with such situations, we developed an approximate estimate of the prior probability based on Monte Carlo simulations. Comparisons on moderately large number of priors suggested that although less accurate, a Monte Carlo estimate of the prior gives results close to the results obtained when calculating the prior exactly (Additional file 2). For larger number of priors, this can not be tested, but experience shows stable results, suggesting that the stochastic nature of the algorithm do not seriously affect the results. With 100 genes and 100 samples and 200 priors, and using 800 for the Monte Carlo prior calculation, it took our method approximately 10 minutes to run 10000 samples on an 64 GB RAM, 16 physical CPU cores compute node.

## Prior information

We will use three sources of prior information: protein-protein interactions, transcription factor binding predictions as well as protein sequence similarity calculations. Gene pairs in these databases have evidence of being functionally related. The strength of the evidence is represented by some type of score for each database. In our MCMC algorithm the prior information is formulated in terms of the prior probability of forcing two genes to belong to the same group. We will use the logit transformation to transform the scores into the unit interval, thus enabling them to be used as a probability measure. Further details are given below for each prior type. There is very little overlap between the prior information databases. In cases where a gene pair is found in more than one database, the highest prior probability will be used.

### Protein interaction data

Protein-protein interactions (PPI) are important for the majority of biological functions. PPI databases contain lists of proteins pairs for which there exist some evidence for interaction. The evidence comes from various types of studies, ranging from high-throughput methods, more traditional low-throughput proteomics studies, as well as in silico predictions based on known interactions [23]. iRefIndex [23] is a consolidated protein interaction database comprising information from nine well-known interaction databases. One of the features of the database is the so-called *lpr* (lowest PMID re-use) score, which is the lowest number of unique interactions that are supported by one of the interaction’s PubMed identifiers (PMID). A low *lpr* indicates that the interaction is more likely to rely on low-throughput methods than on high-throughput methods. As the former is recognized as more reliable than the latter, the *lpr* score can be used as a measure of the reliability of the interaction. We will use a logit transformation taking *x* to 1/(1+*exp*(-*x*)) of the inverse of the lpr score to obtain a probability measure (between zero and one). For example, if the lpr score is one, *p*_*m*_ becomes 0.73, if the lpr score is zero, *p*_*m*_ becomes 0.5.

### Transcription factor bindings

Transcription factors (TF) are proteins that bind to specific DNA sequences, thereby controlling the expression levels of the corresponding genes. Binding preferences of many transcription factors are known and characterized by a sequence binding motif. However, binding affinity does not depend entirely on the match of the sequence to the motif, but will rely also on sequence specific features. Ernst *et al.*[24] developed TF binding predictions, where they first found a general binding preference of the sequences based on 28 local genomic features, including histone modifications, conservation and melting temperature. Then, they combined this general binding preference score with motif information for specific transcription factors to improve prediction of genes bound by the factor. We will make use of these scores, and again, we will apply the logit transformation to transform the scores to values between 0 and 1.

### Protein sequence similarity

Proteins with similar sequences are likely to be functionally related as the proteins may be expressed by paralogous genes (having common ancestry) or by genes that are selected to have the same function. For instance, two homologous proteins can be phosphorylated by the same kinase, thus playing roles in the same signaling pathway. One additional feature of using protein homology data in this setting is that the function of proteins for which the function is unknown can be learned by borrowing information from their protein homologs. We will calculate percent similarity between each of the human proteins in RefSeq using BLAST [25].

## Results

### Simulated data

To evaluate the performance of our method we used simulated gene expression data generated according to [26]. In our study, we used a total of five clusters of genes *C*^∗^=(*C*_1_,…,*C*_5_) with dimension *N* (denoted *d* in [26]) samples. Cluster sizes *n*_*c*_(*c*=1,…,5) were generated from *n*_*c*_∼2×*Poisson*(*λ*). Expression values in cluster *C*_*c*_ were generated using a hierarchical log-normal model as in [26]:

(1) A vector of cluster template for cluster *C*_*c*_ was created with four periods of constant expression of size *m*_1_,*m*_2_,*m*_3_ and *m*_4_. The sizes *m*_*k*_, *k*=1,…,4, was from a uniform distribution such that $\sum_{k}m_{k}=N$ and *m*_*k*_>2. An initial template with constant pattern in four periods was simulated from $\log\;\left(\mu_{k}^{\left(c\right)}\right)∼N\;\left(\mu ,\sigma^{2}\right)$.

(2) Sample variability and gene variability: Sample variability $\sigma_{s}^{2}$ was introduced and the cluster template $T_{j}^{\left(c\right)}$, *j*=1,…,*N*, was generated from $\log\left(T_{j}^{\left(c\right)}\right)∼N\;\left(\log\;\left(\mu_{k}^{\left(c\right)}\right),\sigma_{s}^{2}\right)$. Then for each gene vector *i* in sample *j*, gene variability was added and gene expression values generated as $\log\;\left(x_{\text{ij}}\right)∼N\;\left(\log\;\left(T_{j}^{\left(c\right)}\right),\sigma_{0}^{2}\right)$.

(3) We repeated steps 1 and 2 to generate five clusters. We used parameter values of *μ*=6, *σ*=1, *σ*_*s*_=1, *σ*_0_=1, and *λ*=10.

As in [26], extra variation was introduced to evaluate robustness of clustering methods against potential random errors introduced from experimental procedures, such as sample acquisition, labeling hybridization and scanning. To each element of the log-transformed expression matrix we added a a random error from a normal distribution with mean zero and standard deviation (SD) equal to 0, 1 and 2. In addition, the sample size was varied, using *N* = 10, and 0. For each of these nine scenarios, 50 datasets were generated.

For each dataset, three scenarios for the available prior information were used. In (A), we assumed that no prior information was used. In (B), we assumed priors pairs were available, where 20% where mis-specified, i.e. 20% of the gene pairs had members belonging to different groups. In the last scenario (C), all pairs were assumed to be correctly specified (the members belonged to the same group). Prior values were generated from a uniform U(0.5,1) distribution.

We compared our method with five well-known clustering methods for which a software already exist, namely hierarchical clustering, *k*-means clustering, Partitioning Around Medoids (PAM) [8], Model-based clustering (Mclust) [9] and tight clustering [11]. For all methods except ours, the number of clusters were estimated using the Gap index [27] (our method implicitly searches for the optimal number of clusters). For our method, clusters were inferred by minimizing the posterior expected loss based on the MCMC samples as described in the Methods section. The number of clusters estimated by the GAP index as well as our method is shown by boxplots in Additional file 3: Figure S2. The Rand index, defined as the proportion of concordant gene pairs in two partitions among all possible gene pairs, was used as evaluation measure. Specifically, we used the adjusted Rand index [28], which is standardized to have expected value zero when the partitions are randomly generated and takes maximum value one if two partitions are perfectly identical. Unlike the other methods, tight clustering produces clusters where some genes are not allocated to any cluster. In the calculation of the Rand index, only the allocated genes are considered.

**Figure 1:**
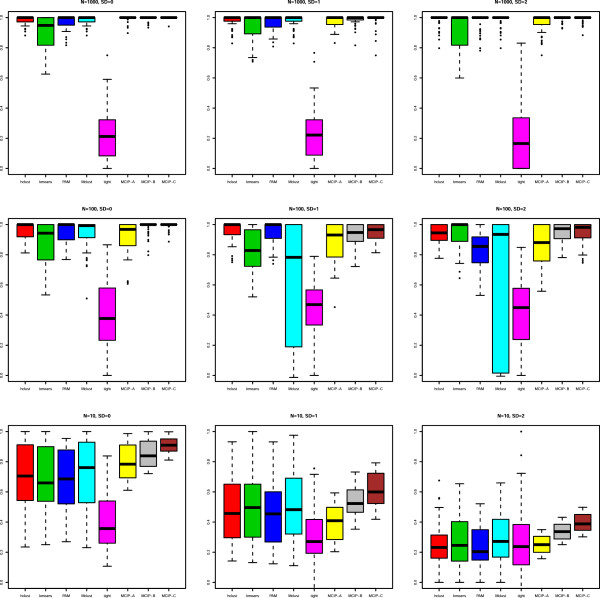
**Results on simulated data.** We have used the simulation scheme proposed by [26] sampling approximately 100 genes per data set. Sample sizes (N) were varied using N = 10, and 0, as well as an extra variation (SD) added to each element in the expression matrix, using SD = 10, and 0. hclust = hierarchical clustering, kmeans = *k*-means clustering, PAM = Prediction Around Medoids, Mclust = model-based clustering, tight = tight clustering, MCIP-A, is our method (MCMC Clustering using Informative Priors), but with no priors used, MCIP-B is our method using priors with 20% of the priors mis-specified, and MCIP-C is our method with all prior pairs correctly specified.

The results are shown in Figure 1. We see that when all pairs are correctly specified (scenario C), our method (MCIP) was at least as good as all other methods, and superior to the other methods for the smallest sample size (N = 10). When 20% of the priors were mis-specified (scenario B), the performance was better than our method without using priors (scenario A), as well as hierarchical clustering, which was overall the second best method. We note that Mclust had a very variable performance, and that tight clustering was performing very poorly for large sample sizes. In order to further investigate the effect of mis-specifications of the priors on model performance, we calculated the adjusted Rand index for increasing proportion of mis-specifications. Additional file 4: Figure S1 shows that about 40% mis-specifications were allowed, in the sense that this corresponded to the use of no prior information. We also note that there was a correspondence between number of estimated clusters (Additional file 3: Figure S2) and performance (Figure 1). Especially for small sample sizes (N = 10), the number of clusters found by maximizing the GAP index, as well as with our method without the use of priors, quite often yielded many more clusters than the true number of clusters (equal to five). This bias was much less evident for our method with the use of priors (MCIP-B and MCIP-C). Additional file 5: Figure S3 shows the performance after fixing the number of clusters to the true number of clusters (five) for all methods except our method, which inherently finds the number of clusters. The figure shows that poor performance, especially seen for tight clustering (N = 100 and N = 1000) and Mclust (N = 100), was not only due to bias in the estimation of number of clusters, as these methods also performed poorly after fixing the number of clusters.

### Heart failure data

We used the data described in [29], consisting of microarray gene expression measurements from fourteen mice subjected to aortic banding and five sham operated mice. Aortic banding leads to increased left ventricular pressure. To compensate for the increased load, gene expression changes occur resulting in myocardial remodeling, involving hypertrophy of cardiomyocytes. Ultimately, the cardiac hypertrophy might lead to development of heart failure. We based our network analysis on the most differentially expressed genes between aortic banding and sham. To find differentially expressed genes we performed *t*-tests between the two groups, using log2 expression values, before multiple testing correction was performed using the method of [30]. We used a false discovery rate (FDR) cut-off of 5%, and among these genes we picked the 400 with largest fold change (up or down). We looked up connections between these genes and assigned prior probabilities for the pairs based on the prior databases described in the previous section. For each of the three prior databases, there were several hundred pairs where both genes were represented in our input-list. As the use of so many priors pairs was too computationally demanding for our method, we picked the 50 top scoring pairs for each of the prior types. We applied our MCMC algorithm using altogether 150000 Monte Carlo samples, with the first 50000 samples used for burn-in (i.e these first samples were discarded and only used to ascertain a stable starting point in the MCMC sampling). We applied parallel tempering as described in Additional file 1. As we here had more than hundred prior pairs, we approximated *P*(***g*** ∣*M*) with the Monte Carlo estimator (1) defined in Additional file 1, using *K*=200, as this value gave stable results within a reasonable computation time. Clusters were inferred by minimizing the posterior expected loss based on the posterior similarity matrix, which was calculated from the collection of each of the 100th MCMC sample after the burn-in period.

**Figure 2:**
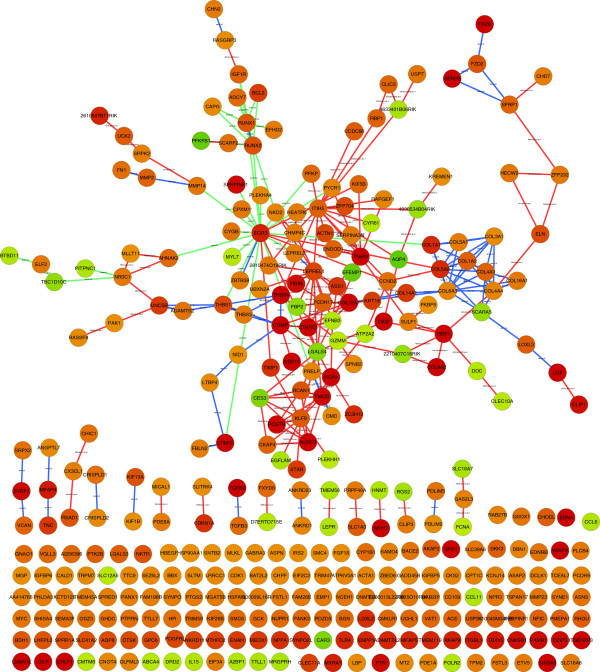
**Heart failure result networks.** Network comprised of prior pairs within the main module. Red node color means upregulated in aorta banding vs sham, green color downregulated. Red edges depict known protein-protein interactions, green edges transcription factor bindings, and blue edges illustrate protein sequence homologies. Text on protein-protein interaction edges denote type of interactions (MI), lowest pmid reuse (lpr) and number of publications (np), text on transcription factor binding edges denote prediction score, and text on protein homology edges denote sequence similarity.

Table 1 summarizes the clusters and Additional file 6: Figure S4 displays the clusters as (fully connected) networks (all nodes in the same cluster are connected) using Cytoscape [31]. There is one one large module of mainly up-regulated genes (aorta banding vs sham), and one smaller module of both up and down-regulated genes. In order to investigate these modules more thoroughly we applied Gene Ontology [32] analysis using the R/Bioconductor package GOstats [33]. Additional file 7 shows the most significantly altered GO categories in each of the modules. The top GO term of the larger module was *extracellular region* (*p* = 3e-34, FDR *q*-value = 5e-31), and many of the other modules were related to this term (*proteinaceous extra-cellular matrix*, *extracellular space*, *extracellular matrix structural constituent*, *collagen*). In the smaller module many GO terms were related to carbohydrate metabolism (*polysaccharide metabolic process*, *carbohydrate biosynthetic process*). Figure 2 contains a subset of the larger network, showing prior pairs occurring within the main module. We also applied our method without the use of priors, as well as *k*-means clustering. For the latter, the Gap index was used to find the number of clusters *K*. Both these methods gave one dominant cluster and two smaller clusters. GO analyses of the main clusters are given in Additional files 8 and 9.

**Table 1:** Summarizing the inferred heart failure clusters

**Cluster**	**Size**	**Upreg. (%)**	**Edges**	**Priors (%)**	**Top three GO terms**
1	315	89.3	98910	0.4	Extracellular region, basement membrane, proteinaceous extracellular matrix
2	85	28.4	7140	0.1	Positive regulation of cell cycle, polysaccharide metabolic process, carbohydrate
					biosynthetic process

Size = number of genes in each cluster, Upreg. (%) = percentage of upregulated genes, Edges = number of edges, Priors (%) = percentage of edges that were prior pairs, and Top three GO terms = the three most significant Gene Ontology terms found by GOstats [33].

### Melanoma cancer data

Metastatic melanoma is a deadly disease while non-metastatic melanoma and other cutaneous tumor types are usually cured with surgical removal of the primary tumors. To find network of genes differentially expressed between metastatic and non-metastatic tumors we used data from [34], which included microarray gene expressions from 47 metastatic and 40 non-metastatic tumor samples of patients with various cutaneous tumors. As for the heart failure data, we used the 400 most differentially expressed genes (based on fold change) for which Benjamini Hochberg FDR <0.05, and found gene pairs in the PPI, the TF and the sequence similarity database where both genes were represented in our input list. We also here approximated *P*(***g*** ∣*M*) with the Monte Carlo estimator (1) of the Additional file 1, using *K*= samples. Again, result clusters were inferred by minimizing the posterior expected loss based on the posterior similarity matrix calculated from each th of 150K MCMC samples generated after a burn-in period of 50K samples.

**Figure 3:**
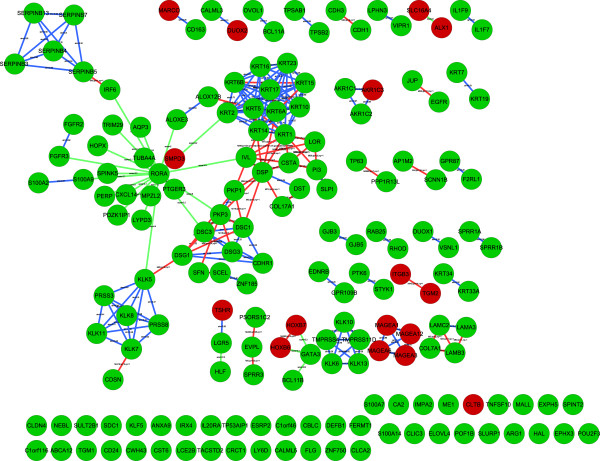
**Melanoma cancer result networks.** Prior pairs within the largest module. Red node color means upregulated in metastatic melanoma, green color downregulated. Red edges illustrate known protein-protein interactions, green edges transcription factor bindings, and blue edges protein sequence homologies. Text on protein-protein interaction edges denote type of interactions (MI), lowest pmid reuse (lpr) and number of publications (np), text on transcription factor binding edges denote prediction score, and text on protein homology edges denote sequence similarity.

Table 2 summarizes the clusters/modules and Additional file10: Figure S5 shows the modules as (fully connected) networks. Figure 3 shows prior pairs within each module. The top ten GO categories from GOstats analysis on each module are shown in Additional file 11. We note that the top three GO categories of the largest module were *epidermis development* (*p* =6e-32, FDR *q*-value =8e-29), *cornified envelope* (*p* =2e-18, FDR *q*-value =3e-15) and *keratinization* (*p* =1e-14, FDR *q*-value =3e-15). We also applied our method without the use of priors and *k*-means clustering to the melanoma data. GO analyses of the main clusters are given in Additional files 12 and 13.

**Table 2:** Summarizing the inferred melanoma cancer clusters

**Cluster**	**Size**	**Upreg. (%)**	**Edges**	**Priors (%)**	**Top three GO terms**
1	130	2.3	16770	1.3	Epidermis development, cornified envelope, keratinization
2	25	4.0	600	0.0	Androgen biosynthetic process, extracellular region, desmosome
3	28	3.6	756	1.7	Forebrain morphogenesis, response to vitamin A, negative regulation of neuron maturation
4	25	0.0	600	1.2	Keratinization, peptide cross-linking, desmosome
5	17	52.9	272	1.8	Testosterone 16-alpha-hydroxylase activity, negative regulation of Rho GTPase activity,
					negative regulation of epidermal growth factor-activated receptor activity
6	13	61.5	156	1.9	Alcohol sulfotransferase activity, CDP-diacylglycerol biosynthetic process, embryonic
					hindgut morphogenesis
7	13	53.8	156	1.3	Regulation of mesonephros development, negative regulation of cell proliferation involved
					in mesonephros development, negative regulation of fibroblast growth factor receptor
					signaling pathway involved in ureteric bud formation
8	12	0.0	132	0.0	Glutamate dehydrogenase (NAD+) activity, glutamate dehydrogenase [NAD(P)+] activity,
					negative regulation of myelination
9	12	16.7	132	1.5	Serine-type endopeptidase activity, serine hydrolase activity, peptidase activity, acting on
					L-amino acid peptides
10	10	50.0	90	0.0	Adherens junction organization,interleukin-1 Type I receptor binding, dihydrotestosterone
					17-beta-dehydrogenase activity
11	10	0	90	1.1	N-acylglucosamine 2-epimerase activity, osteoclast proliferation, alkaloid catabolic process
12	10	30.0	90	0.0	Middle ear morphogenesis, vagus nerve morphogenesis, activation of phospholipase
					D activity by G-protein coupled receptor protein signaling pathway
13	11	9.1	110	0.9	Scavenger receptor activity, negative regulation of collateral sprouting of intact axon
					in response to injury, regulation of cell adhesion
14	11	0.0	110	0.9	Positive regulation of cell development, regulation of cell morphogenesis involved in
					differentiation, argininosuccinate synthase activity
15	12	16.7	132	0.0	Pharynx development, cellular response to external biotic stimulus, nucleus
16	9	0.0	72		1.4 Alpha-dystroglycan binding, isopeptide cross-linking via N6-(L-isoglutamyl)-L lysine,
					positive regulation of arachidonic acid secretion
17	7	100.0	42	14.3	Vascular transport, interleukin-1 Type II blocking receptor activity, Golgi cis cisterna
18	6	66.7	30	3.3	NLRP1 inammasome complex, 17-alpha,20-alpha-dihydroxypregn-4-en-3-one
					dehydrogenase activity, androsterone dehydrogenase (B-specific) activity

Size = number of genes in each cluster, Upreg. (%) = percentage of upregulated genes, Edges = number of edges, Priors (%) = percentage of edges that were prior pairs, and Top three GO terms = the three most significant Gene Ontology terms found by GOstats [33].

### Method evaluation

In order to evaluate our method we made use of literature reported interactions that occurred in abstracts of articles labeled with the Medical Subject Headings (MeSH, http://www.nlm.nih.gov/mesh/) term *left ventricular hypertrophy* for the heart failure clusters (summarized in Table 1 and displayed in Additional file 6: Figure S4) and the MeSH term *melanoma* for the melanoma clusters (summarized in Table 2 and displayed in Additional file 10: Figure S5). We applied gene pairs with *p*-values smaller than 5% only, using the method described in [35]. We will refer to these interactions as "true" interactions. The application of our method together with minimization of the posterior expected loss led to an inferred clustering. By considering whether the genes of each possible gene pair occurred in the same group or not in the inferred clustering, we were able to calculate the sensitivity (proportion of gene pairs in the literature network also occurring together in the same group in the inferred clustering), the specificity (proportion of gene pairs not in the literature network that were also not in the same clustering group), the positive predictive value (proportion of genes occurring in the same group also occurring in the literature network). From the sensitivities and specificities the Area Under Curve (AUC) was also calculated. Table 3 shows performance measures for the heart failure and the melanoma data, respectively, using our method both with and without priors, and compared to the results of *k*-means clustering. For *k*-means clustering the optimal number of clusters was found using the Gap index.

**Table 3:** **Comparing performance of our method (MCIP) with and without the use of priors to** ***k*-means clustering**

	**Heart failure data**					**Melanoma data**				
	**Sens.**	**Spec.**	**PPV**	**AUC**	***K***	**Sens.**	**Spec.**	**PPV**	**AUC**	***K***
MCIP with priors	0.62	0.68	0.023	0.65	2	0.67	0.6	0.030	0.64	18
MCIP without priors	0.61	0.64	0.020	0.62	3	0.68	0.53	0.024	0.60	20
Kmeans	0.52	0.65	0.019	0.59	3	0.70	0.48	0.021	0.58	10

The performances are obtained by evaluating the result clusters against a literature based reference network comprising pairs of genes co-cited in Pubmed articles with the Medical Subject Headings (MeSH) left ventricular hypertrophy (for the heart failure data) and melanoma cancer (for the melanoma cancer data). Sens.=sensitivity, Spec. = specificity, PPV = Positive predictive value, AUC = Area under receiver operator curve, and *K* = number of clusters, which is automatically found by our method, and by using the Gap index for *k*-means clustering.

## Discussion

Using simulated data we compared our method to established clustering methods as *k*-means and hierarchical clustering. When no prior information is provided our method did not offer any gains over other standard approaches, except for the N = 10, SD = 0 regime. When most priors were correctly specified (80% or 100%), our method was as least as good as the best-performing established methods for large samples, and superior to the same methods for small sample sizes. We believe that the majority of the priors will be correctly specified in most cases. The reason for this is that there will usually exist so many prior pairs that one would restrict oneself to only those with the strongest evidence. Of course, a previously shown connection might still not be real in the current situation, e.g. if the connection has been discovered in a different tissue type than the one under study. However, this problem will be limited if one is analyzing a set of genes differentially expressed between two conditions, as many of these will be co-expressed, and thus also correlated.

The Gene Ontology analysis of the heart failure network showed that many of the top ranked GO categories for the larger module were related to the extracellular matrix. Awareness has emerged regarding the extracellular matrix changes taking place in myocardial remodeling [36,37]. When investigating Figure 2, we see that the larger module contains many collagens (type I alpha 1, type III alpha 1, type IV alpha 1, type V alpha 1, type VI alpha 1, type XII alpha 1, type XIV alpha 1 and type XVI a), which are important structural components of the extracellular matrix. The figure shows that these molecules were primarily connected by protein sequence homology (blue lines). Moreover, other molecules thought to be parts of the extracellular matrix were identified in the same module (thrombospondin 3 and 4, fibromodulin, versican, biglycan, fibronectin, elastin). For example, fibronectin has been demonstrated to play a role in the organizing of collagen type I [38]. The module also contained enzymes and hormonal factors known to process the extracellular matrix structural proteins (matrix metallopeptidase 2, transforming growth factor beta 2 and beta 3, latent transforming growth factor beta binding protein 2, lysyl oxidase, TIMP metallopeptidase inhibitor 1). For instance, matrix metallopeptidase 2 is known to be important for the breakdown of collagen type I [39]. Finally, the extracellular matrix related transcription factor early growth response 3 was a hub in this module, indicating a particular importance in this pathological process. Altered metabolism has been described in remodeled and failing hearts. E.g. reduced fatty acid oxidation and an increase in glucose utilization have been reported [40-42]. The smaller module contained several genes involved in carbohydrate metabolic processes (6-phosphofructo-2-kinase, phosphorylase kinase gamma 1, aldolase B fructose-bisphosphate, fructose bisphosphatase 2). Taken together, the two modules confirm regulatory and signaling events and structural alterations known to be involved in myocardial remodeling. We believe that the complete result network, shown in Additional file 6: Figure S4, contains many other important, not yet discovered, interactions. However, a detailed investigation of all these connections is beyond the scope of this paper.

The melanoma network was derived from the data of Riker *et al.*[34]. As the present paper represents a reanalysis of the data, the outcome of an analysis with similar input data should be expected to generate somewhat similar output data. The main difference in approach is that while the Riker paper primarily performs a direct Gene Ontology analysis, the current approach attempts to combine multiple sources of information prior to performing any functional analysis. In this way, the resultant network modules will represent subnets of relevance with respect to the combined prior information, thus presenting local information clusters with higher likelihood of coherence in function for each cluster. Riker *et al.* observed differentially expressed genes involved in keratinocyte differentiation and epidermal development, including loricrin (LOR), involucrin (IVL), keratin 5 (KRT5) and plakophilin 1 (PKP1), suggesting a loss of epidermal characteristics, in highlighting the desquamation process. In our largest module (module 1) we found many genes related to keratinocyte differentiation and epidermal development (see Additional file 11, Table 1). When looking at Figure 3, where gene pairs with prior information within each module were connected, we note that keratins and kallikreins were connected through a set of interacting genes that included desmocollin 1 and 3 (DSC1 and DSC3), desmoglein 1 and 3 (DSG1 and DSG3), desmoplakin (DSP) and corneodesmosin (CDSN). Many of the genes in this path are related to corneodesmosomes (KLK5 and KLK7: kallikrein 5 and 7, DSG1, DSG3, DSC1, DSC3, DSP, IVL, LOR and keratin 10). Corneodesmosomes are located at the junctional structures that mediate corneocyte cohesion [43], underlining keratinocyte dedifferentiation. The individual connections in this path are supported by previous findings; Caubet *et al.*[43] showed that KLK5 and KLK7 degrade the desmosomal proteins DSC1 and DSG1. Smith *et al.*[44] showed that when epidermal cells differentiate, DSC1 and DSG1 are bound by PKP1 and DSP, thereby enhancing anchorage to keratin intermediate filaments. Interactions between plakophilins, desmoplakins and keratins have also been shown by other authors, e.g. [45-47]. Interestingly, the transcription factor RAR-related orphan receptor A (RORA) was downregulated, and placed as a hub in this module. RORA has recently been indicated as a breast tumor suppressor [48], as well as having a role in keratinocyte differentiation [49]. Taken together, Figure 3 suggests that the loss of epidermal characteristics seen in the metastatic individuals is mediated by a downregulation of the RORA transcription factor.

When comparing the GO analysis of the main clusters from our method with the use of priors (Additional file 7 and 11) to the GO analysis of the main clusters using our method without priors (Additional files 8 and 9) and *k*-means clustering (Additional files 9 and 13), we note that many of the same GO categories were showing up. But the *p*-values were overall much smaller for our method with the use of priors, than the other two, indicating that our method has better sensitivity of finding functionally related gene groups. We note that the path found in the melanoma data linking the RORA transcription factor to the keratins via the kallikreins and the desmosomal proteins, would not have been discovered by our method without priors or *k*-means clustering, as none of the these methods gave clusterings where all these gene subgroups were contained in one cluster.

Method evaluation using the literature-derived network showed that adding prior knowledge yielded improved AUC. The AUC was improved from 0.62 to 0.65 for the heart failure data and from 0.6 to 0.64 for the melanoma data. The positive predicted values (PPV) were also improved (0.02 to 0.023 for the heart failure data and 0.024 to 0.03 for the melanoma data). We note that these values are very small, which is because there are very few gene pairs with co-citation (only around one in a hundred of all possible pairs) compared to the number of gene pairs clustered together (more than half of the pairs for the heart failure data, for instance). It is important to be aware that the possibility of improvement is limited by the degree of overlap between the list of pairs in the prior interaction databases and list of pairs in the literature reference network. Many of the pairs in the literature databases were not found in the interaction databases. In fact this was the case for about 90% for these data. Such interactions are, when evaluated on the literature network, treated as false positives, but might of course be true positives. Of the 90% of the pairs in the literature network that were not in the prior databases, the evidence for grouping the genes together was based solely on the correlation in the microarray data. Another limitation of the literature network is that it itself might contain false positives. Genes that are mentioned in the same PubMed abstract are not necessarily connected.

We have here focused on finding groups of connected genes, and not on discovering direct interactions. Direct interactions are often inferred using Bayesian networks (BN). As mentioned in the Background section, the BN formalism allows for incorporation of prior knowledge, and BN methods for genomic data has indeed been proposed. However, constructing large-scale networks using BN methodology is very difficult as the number of possible configurations is about exponential in the number of genes. Formally this has been shown to be an NP-Complete problem. By instead focusing on groups, we heavily reduce the number of possible configurations. Thus, our method can handle many more genes than BN methods. This is relevant, as microarray data may involve several hundred regulated genes. One way of using our method is to apply it as an initial step prior to a BN analysis; If the number of genes is too large to apply BN methodology to the full set, our method can be used to first find smaller-sized, independent sets of genes, followed by separate BN analysis on each of the subsets. The result of such an analysis will be similar to the networks shown in Figures 2 and 3, where prior pairs within each cluster are shown. We believe that the connections shown in Figures 2 and 3 are reliable and robust, as they display connections between genes that are co-regulated and that have a previously shown connection. Using BN on each cluster could lead to an improvement, as novel interactions can be detected based on strong correlations in the data, and is one possibility for future methodological development.

Along with the development of the primarily data-driven methods, various methods focusing more on the prior information have been proposed, e.g. [5,50,51]. In these approaches, the authors made use of the observed changes in the experimental data, e.g. the most differentially regulated genes, while the interactions among these genes were derived from prior information only. For example, Huang *et al.*[5] applied the so-called Prize-collecting Steiner tree to select nodes and interactions. As we speak, the prior knowledge about molecular interactions is rapidly increasing. For instance, the data generated by the large-scale consortium project Encode [52] represent huge possibilities for extending the source of prior knowledge about transcriptional regulation. Obviously, methods enabling the use of prior knowledge when constructing genetic networks will increase their relevance in line with this development.

## Conclusions

We have presented a novel method for finding modules of interacting molecules, representing biological pathways or processes, based on microarrays or other transcriptomic data. The method is a clustering approach, which is a commonly used technique for analyzing transcriptomic data. Unlike previous approaches using Bayesian statistics to infer clusters, e.g. [9,10], we used informative (as opposed to non-informative) priors. The prior information was assumed to be in form of pairs of connected genes, along with a connection score, as this enabled us to capture a lot of relevant prior information, like protein-protein interactions, transcription factor binding information and protein sequence similarity measurements. Using simulated data our method showed improved ability of identifying correct groups compared to traditional clustering, especially for small sample sizes, and for situations where most priors were correctly specified. When applying the method to real-world microarray data in heart failure and melanoma cancer we found clusters overlapping with known pathogenic processes, but where subnetworks of prior pairs within each cluster pointed to new connections extending beyond the classical disease pathways.

## Availability

The method is implemented in C++ and R and is available from: http://folk.uio.no/trondr/gene_corr, as well as a tool in the Galaxy [53] based Genomic HyperBrowser [54], see http://hyperbrowser.uio.no/dev2 under assorted tools →MCMC gene corr. The data sets supporting the results of this article are available from the Gene Expression Omnibus repository: http://www.ncbi.nlm.nih.gov/geo/query/acc.cgi?acc=GSE36074 (heart failure data) and http://www.ncbi.nlm.nih.gov/geo/query/acc.cgi?acc=GSE7553 (melanoma cancer data).

## Competing interests

The authors declare that they have no competing interests.

## Authors’ contributions

SN and TR developed the methodology. SN, TR and VN implemented the software. SN, TR, TC, VN, JB, BS and TT generated and collected data used in this paper. SN, TR, TC, JB, GC and EH analyzed the results. SN, TR, JB, GC and EH wrote the article. All authors participated in discussions, read and approved the final manuscript.

## Supplementary Material

Additional file 1 **Calculations and parallel tempering description.** Calculations of expressions used in the MCMC algorithm and description of the use of parallel tempering. Click here for file

Additional file 2 **Monte Carlo estimation of prior.** Evaluation of the precision of the Monte Carlo estimates of the prior compared to the exact calculation. Click here for file

Additional file 3 **Figure S2.** Number of estimated clusters. GAP index is used for all methods except ours, which inherently finds the number of clusters. hclust = hierarchical clustering, kmeans = *k*-means clustering, PAM = Prediction Around Medoids, Mclust = model-based clustering, tight = tight clustering, MCIP-A, is our method (MCMC Clustering using Informative Priors), but with no priors used, MCIP-B is our method using priors with 20% of the priors mis-specified, and MCIP-C is our method with all prior pairs correctly specified. Click here for file

Additional file 4 **Figure S1.** Investigation of the effect of mis-specified priors on model performance. The figure shows boxplots of adjusted Rand index values for 50 simulated datasets using the simulation set-up described in the Results section, using extra variation SD of 0, 1 and 2, and number of individuals *N* of 10, and 0. Click here for file

Additional file 5 **Figure S3.** Evaluation of performance in terms of adjusted Rand index following the simulation scheme of [26]. The simulation setup is identical to the one generating Figure 1, except that for this figure the number of clusters where fixed to the true number of clusters (five). hclust = hierarchical clustering, kmeans = *k*-means clustering, PAM = Prediction Around Medoids, Mclust = model-based clustering, tight = tight clustering, MCIP-A, is our method (MCMC Clustering using Informative Priors), but with no priors used, MCIP-B is our method using priors with 20% of the priors mis-specified, and MCIP-C is our method with all prior pairs correctly specified. Click here for file

Additional file 6 **Figure S4.** The results of applying our method to the most differentially expressed genes between aorta banding and sham in the microarray heart failure data. Red node color means upregulated in aorta banding vs sham, green color downregulated. Click here for file

Additional file 7 **GO results MCIP with priors, heart failure data.** Results of Gene ontology analysis using GOstats [33] of heart failure clusters found using our method with priors. Click here for file

Additional file 8 **GO results MCIP without priors, main cluster, heart failure data.** Results of Gene ontology analysis of main heart failure cluster found using our method without priors. Click here for file

Additional file 9 **GO results Kmeans clustering, main cluster, heart failure data.** Results of Gene ontology analysis of main heart failure cluster found using Kmeans clustering. Click here for file

Additional file 10 **Figure S5.** The results of applying our method to the most differentially expressed genes between metastatic and non-metastatic melanoma cancer patients. Red node color means upregulated in metastatic melanoma, green color downregulated. Click here for file

Additional file 11 **GO results MCIP with priors, melanoma data.** Results of Gene ontology analysis of melanoma clusters found using our method with priors. Click here for file

Additional file 12 **GO results MCIP without priors, main cluster, melanoma cancer data.** Results of Gene ontology analysis of main melanoma cluster found using our method without priors. Click here for file

Additional file 13 **GO results Kmeans clustering, main cluster, melanoma cancer data.** Results of Gene ontology analysis of main melanoma cluster found using Kmeans clustering. Click here for file
