# Supplementary material for: Identifying pathogenic processes by integrating microarray data with prior knowledge
Source: BMC Bioinformatics. 2014 Apr 24;15:115. doi: 10.1186/1471-2105-15-115 (PMC4006456; doi:10.1186/1471-2105-15-115)
Supplement: Additional file 1 — Calculations and parallel tempering description. Calculations of expressions used in the MCMC algorithm and description of the use of parallel tempering. [file 1471-2105-15-115-S1.PDF]

Additional file 1

Prior Calculations

When the number of prior pairs q in M increases, doing the calculation in Eq. (2) increases exponentially, as the number of possible configurations of X is 2^q. To avoid exponential computational cost with increasing prior knowledge, a Monte Carlo estimate of the prior can be performed. If a maximum of K operations is wanted per prior calculation, then if $2^q \leq K$, we calculate equation (2) directly. If $2^q > K$, we sample X k times from the probability distribution $\prod_{m=1}^{q} p_m^{X_m}(1-p_m)^{1-X_m}$. This can be done simply by drawing each X_m, with outcome $X_m = 1$ having probability p_m and outcome $X_m = 0$ having probability $(1-p_m)$. For K such X samples, $(X^{(1)}, \ldots, X^{(K)})$, the Monte Carlo estimate of the prior probability of grouping g will be

$$\widehat{P(\boldsymbol{g}|M)} \approx \frac{1}{K}\sum_{i=1}^{K} \frac{I(k \leq n, (i_m, j_m) \in \boldsymbol{g}\ \forall\ m|X_m^{(i)} = 1)}{(n-x^{(i)})N(n-x^{(i)}, k_g)}. \quad (1)$$

Such a Monte Carlo calculation will be an unbiased estimate for the real prior probability, while keeping the computational cost down to a fixed level K.

Parallel tempering

In our application, where the parameter space is made out of discreet groupings, there may easily be two groupings with high likelihood and prior where there is no way to reach one such grouping from the next via one single change. Thus, intermediate groupings with lower probability mass would in such a case need to be traversed by the MCMC algorithm before both high probability mass groupings would be explored. Parallel tempering (Geyer, 1991) provides one way of dealing with such problems. In addition to sampling from the probability distribution one is interested in, one can also sample from other distributions in parallel. These other distributions could be smoothed versions of the distribution of interest. In parallel tempering, one makes these smoothed distributions as $f_{new}(\theta) \propto \mathrm{e}^{-log(g(\theta))/T}$ with $g(\theta) \propto f(D|\theta) \propto f(\theta)f(D|\theta)$ with $T \geq 1$. The case $T = 1$ constitutes the distribution of interest. The higher temperature distributions will have a lower difference between peaks and valleys in the probability landscape, and thus will have improved mixing, compared to the distribution of interest. In parallel tempering, one allows for the algorithm to propose, with a fixed probability, that the parameter state of two chains should be switched. This switch is accepted with the Metropolis-Hastings acceptance rate for this switch. Thus the mixing that may occur in high temperature chains, may allow the original chain to move from one high probability region to another without having to step through the less probable regions of parameter space. In our implementation, we allowed for an arbitrary number of chains, $i \in \{1, \ldots, n_T\}$, to be run in parallel, with $T_i > T_{i-1}$ for $i > 1$ and $T_1 = 1$. Switches were only allowed for neighboring chains $i-1$ and i. In the specific analysis, we used $n_T = 10$ chains with $T = \{1, 1.3, 1.7, 2.2, 3, 4, 5, 6, 7, 8)\}$. The temperatures were chosen so as to get the number of accepted switches relatively high between all neighboring chains.

References

. Geyer, C (1991) Markov-Chain Monte-Carlo Maximum-Likelihood, *In: Keramidas, EM, editor, Computing Science and Statistics. Interface Foundataion North America*, pp. 156-163.
