# Supplementary material for: Identifying pathogenic processes by integrating microarray data with prior knowledge
Source: BMC Bioinformatics. 2014 Apr 24;15:115. doi: 10.1186/1471-2105-15-115 (PMC4006456; doi:10.1186/1471-2105-15-115)
Supplement: Additional file 2 — Monte Carlo estimation of prior. Evaluation of the precision of the Monte Carlo estimates of the prior compared to the exact calculation. [file 1471-2105-15-115-S2.PDF]

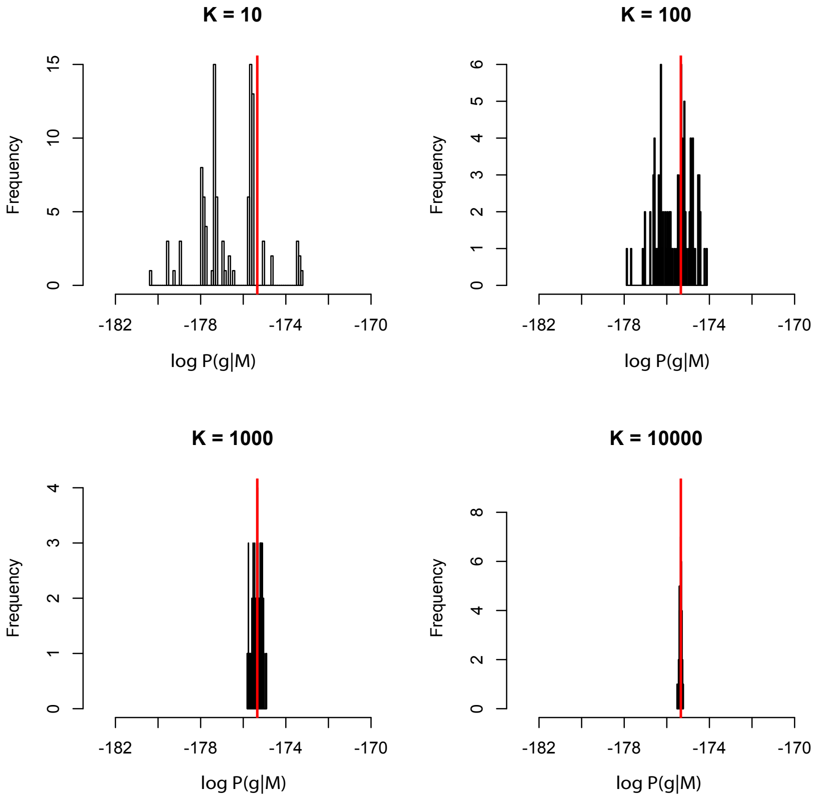

Precision of Monte Carlo estimator of prior

In order to examine the precision of the Monte Carlo estimate of the prior probability of a grouping, $P(g|M)$, we performed multiple Monte Carlo estimates and compared with the exact probability. We assumed there were ten equally sized groups, and drew alltogehter ten pairs from these groups, where each pair was assigned probability $p_m = 0.8$. For numbers of Monte Carlo samples, K, equal to 10, 100, 1000 and 10000, we performed 100 estimations of $P(g|M)$ and drew histograms of the estimates and compared with the exact probability (red vertical line).
