# Supplementary figures and images for: Identifying pathogenic processes by integrating microarray data with prior knowledge
Source: BMC Bioinformatics. 2014 Apr 24;15:115. doi: 10.1186/1471-2105-15-115 (PMC4006456; doi:10.1186/1471-2105-15-115)

**N=1000, SD=0**

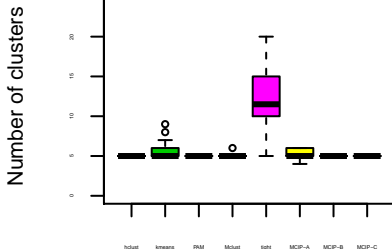

**N=1000, SD=1**

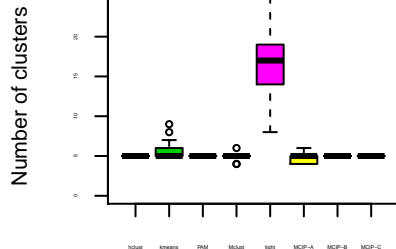

**N=1000, SD=2**

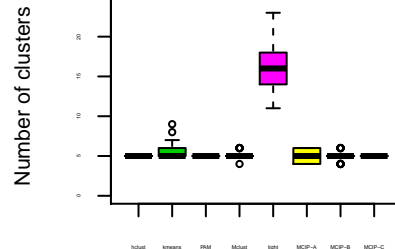

**N=100, SD=0**

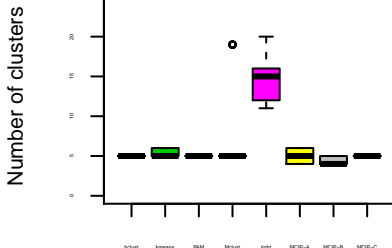

**N=100, SD=1**

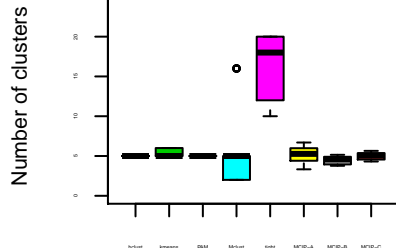

**N=100, SD=2**

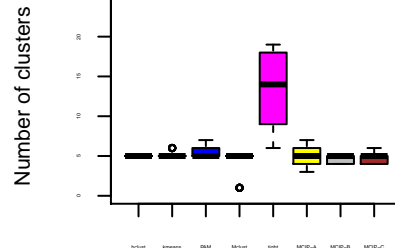

**N=10, SD=0**

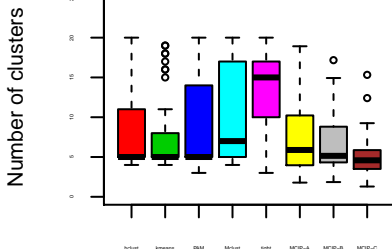

**N=10, SD=1**

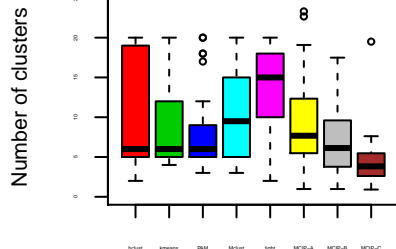

**N=10, SD=2**

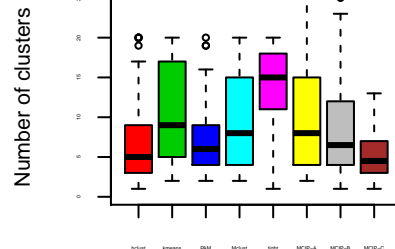

Supplement: Additional file 3 — Figure S2. Number of estimated clusters. GAP index is used for all methods except ours, which inherently finds the number of clusters. hclust = hierarchical clustering, kmeans = k-means clustering, PAM = Prediction Around Medoids, Mclust = model-based clustering, tight = tight clustering, MCIP-A, is our method (MCMC Clustering using Informative Priors), but with no priors used, MCIP-B is our method using priors with 20% of the priors mis-specified, and MCIP-C is our method with all prior pairs correctly specified. [file 1471-2105-15-115-S3.PDF]

N=1000, SD=0

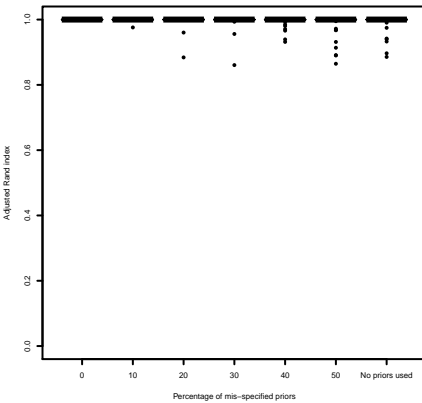

N=1000, SD=1

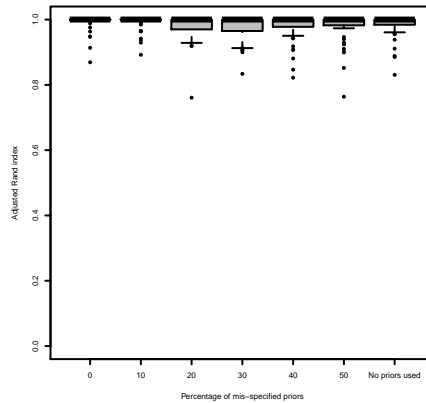

N=1000, SD=2

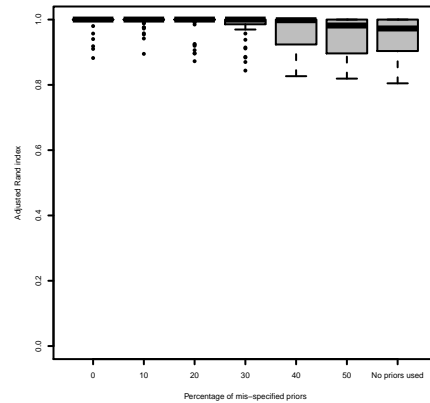

N=100, SD=0

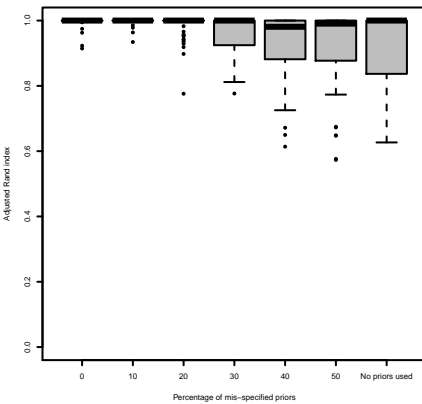

N=100, SD=1

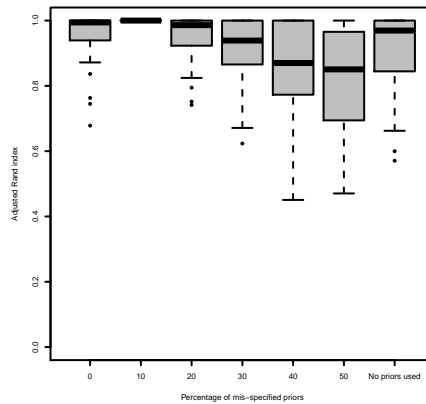

N=100, SD=2

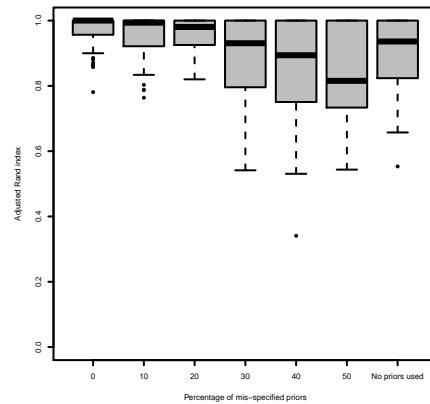

N=10, SD=0

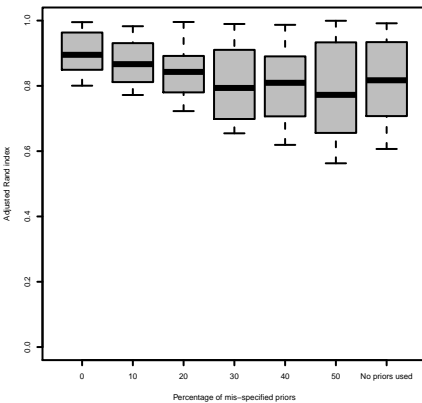

N=10, SD=1

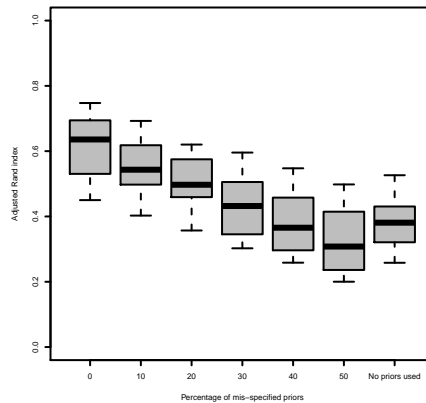

N=10, SD=2

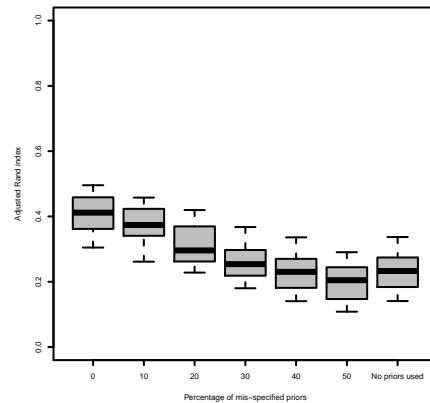

Supplement: Additional file 4 — Figure S1. Investigation of the effect of mis-specified priors on model performance. The figure shows boxplots of adjusted Rand index values for 50 simulated datasets using the simulation set-up described in the Results section, using extra variation SD of 0, 1 and 2, and number of individuals N of 10, and 0. [file 1471-2105-15-115-S4.PDF]

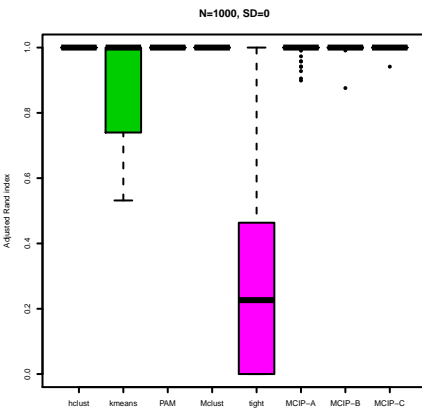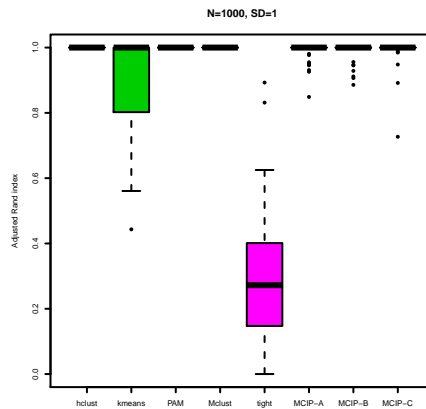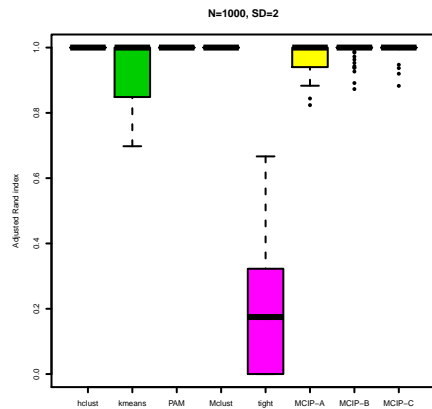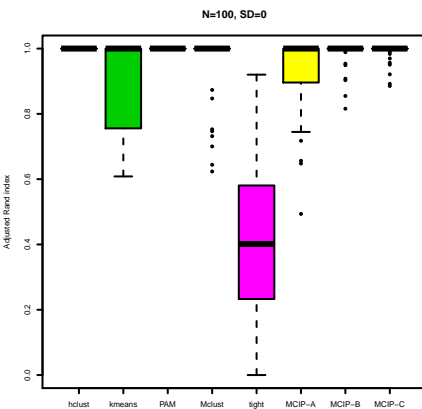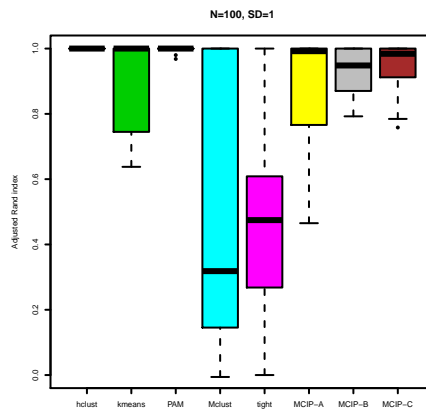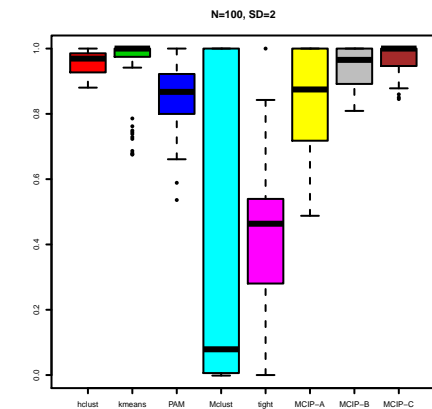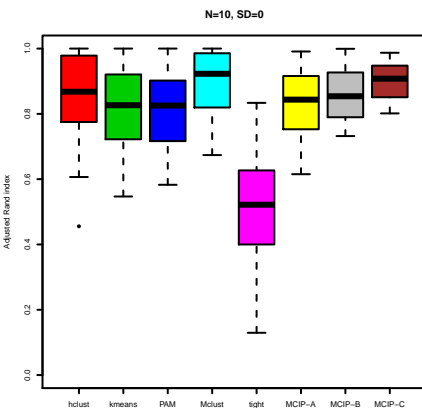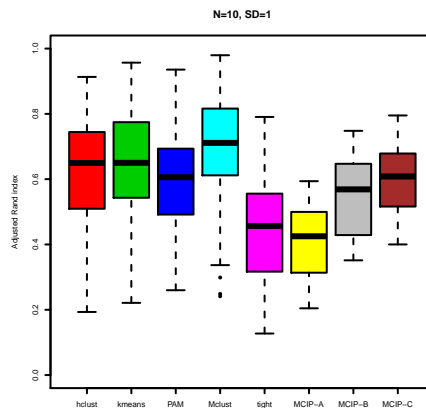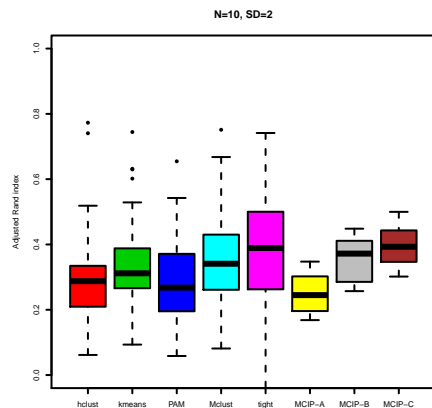

Supplement: Additional file 5 — Figure S3. Evaluation of performance in terms of adjusted Rand index following the simulation scheme of [26]. The simulation setup is identical to the one generating Figure 1, except that for this figure the number of clusters where fixed to the true number of clusters (five). hclust = hierarchical clustering, kmeans = k-means clustering, PAM = Prediction Around Medoids, Mclust = model-based clustering, tight = tight clustering, MCIP-A, is our method (MCMC Clustering using Informative Priors), but with no priors used, MCIP-B is our method using priors with 20% of the priors mis-specified, and MCIP-C is our method with all prior pairs correctly specified. [file 1471-2105-15-115-S5.PDF]

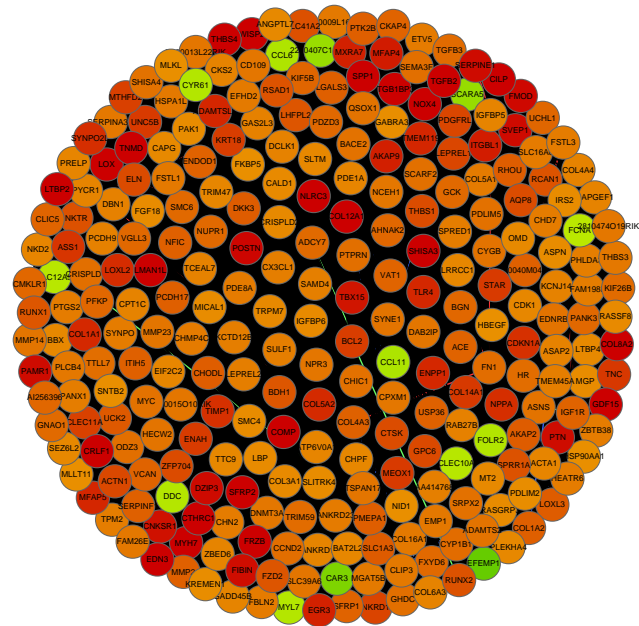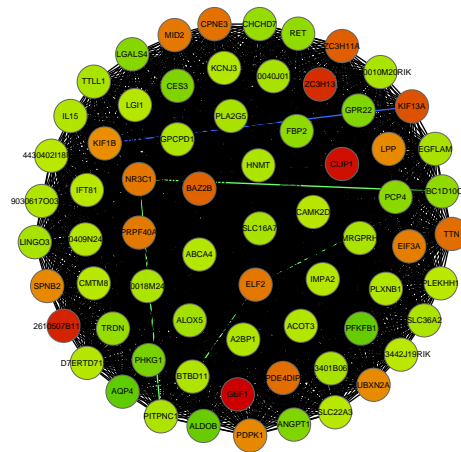

Supplement: Additional file 6 — Figure S4. The results of applying our method to the most differentially expressed genes between aorta banding and sham in the microarray heart failure data. Red node color means upregulated in aorta banding vs sham, green color downregulated. [file 1471-2105-15-115-S6.PDF]

A

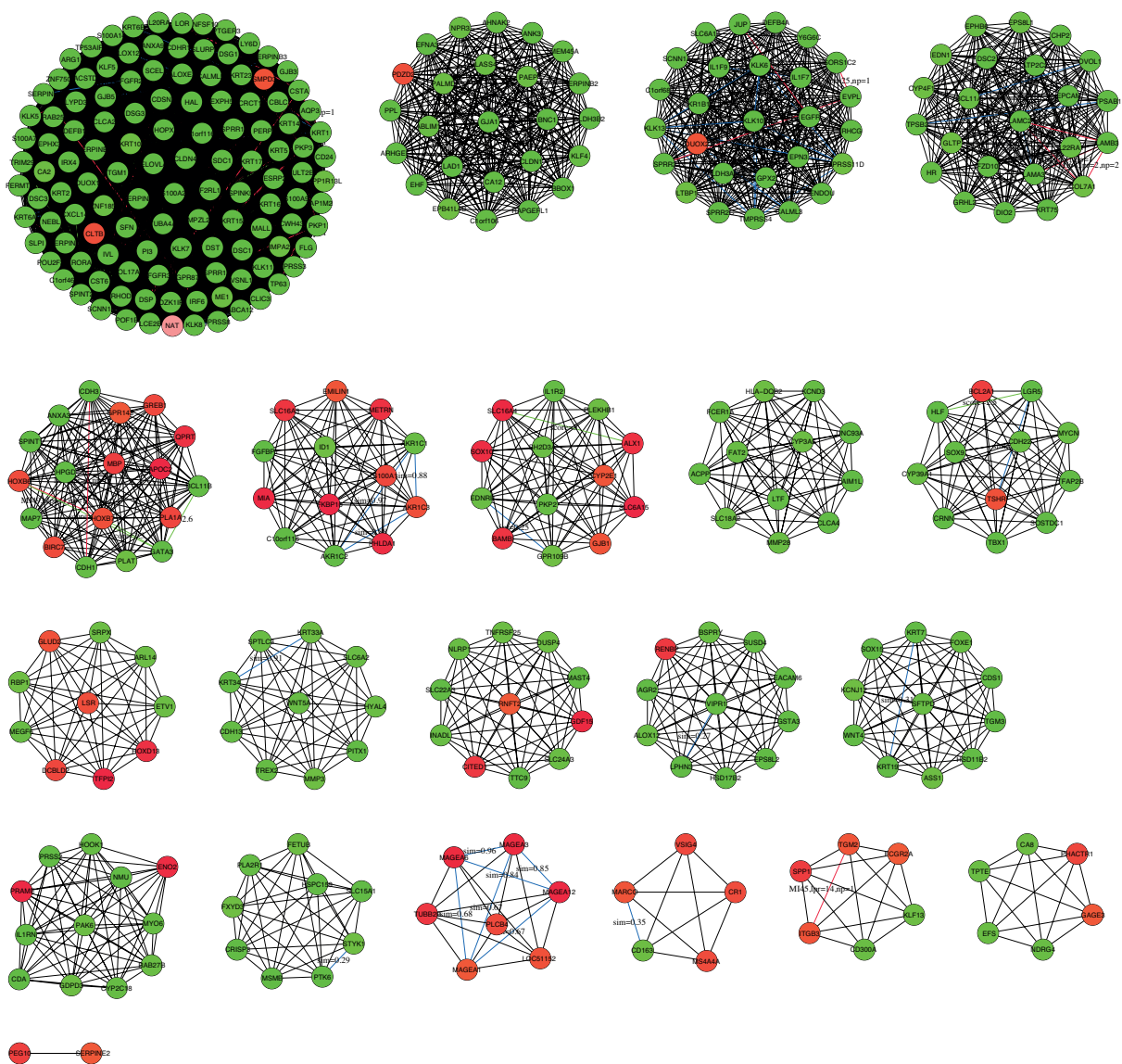

Supplement: Additional file 10 — Figure S5. The results of applying our method to the most differentially expressed genes between metastatic and non-metastatic melanoma cancer patients. Red node color means upregulated in metastatic melanoma, green color downregulated. [file 1471-2105-15-115-S10.PDF]
