# Supplementary material for: Identifying pathogenic processes by integrating microarray data with prior knowledge
Source: BMC Bioinformatics. 2014 Apr 24;15:115. doi: 10.1186/1471-2105-15-115 (PMC4006456; doi:10.1186/1471-2105-15-115)
Supplement: Additional file 7 — GO results MCIP with priors, heart failure data. Results of Gene ontology analysis using GOstats [33] of heart failure clusters found using our method with priors. [file 1471-2105-15-115-S7.PDF]

Table 1: Genes in cluster: 2210407C18RIK, 3110040M04RIK, ACTN1, ADAMTSL2, AKAP2, AKAP9, ANKRD11, AQP8, ASAP2, ASS1, BCL2, BCL2L1, BDH1, BGN, BID, BNC2, CAR3, CCDC80, CCND2, CDKN1A, CHD2, CHODL, CILP, CLEC11A, CNKSR1, COL12A1, COL14A1, COL15A1, COL1A1, COL1A2, COL3A1, COL4A1, COL4A3, COL5A1, COL5A2, COL8A1, COL8A2, COMP, CRLF1, CTGF, CTHRC1, CTSK, CUEDC1, CYTH3, DDAH1, DIO2, DKK3, DOK1, DZIP3, EDN3, EFEMP1, EGR2, EGR3, ELN, ENAH, ENDOD1, ENPP1, FBLN1, FIBIN, FMOD, FN1, FRZB, FZD2, GDF15, GPC6, GRB10, IRS2, ITGA5, ITGB1BP3, ITGBL1, ITIH5, KCNA1, KDM5A, KRT18, LAMA4, LAMC1, LEPREL1, LGALS3, LHFPL2, LMAN1L, LOX, LOXL2, LOXL3, LRP1, LTBP2, LUZP1, MEOX1, MFAP4, MFAP5, MMP2, MTAP1B, MTHFD2, MXRA7, MYC, MYH7, NFIC, NID1, NKTR, NLRC3, NOX4, NPPA, PAMR1, PCDH17, PDGFRL, PDLIM7, PKD1, PMEPA1, POSTN, PRELP, PTK2B, PTN, PTPRF, RBP1, RCAN1, RHOD, RHOU, RSAD1, RTN4, RUNX1, RUNX2, SCARA5, SERPINE1, SERPINF1, SFRP2, SFRS2IP, SHISA3, SLC1A3, SLC41A2, SMAD1, SPP1, SPRR1A, STAR, SVEP1, SYNPO2L, TBX15, TGFB2, THBS1, THBS4, TIMP1, TIMP2, TIMP3, TLR4, TMEM119, TNC, TNMD, UCK2, UNC5B, USP36, WISP2, WNK1, ZFP704, ZYX

	GO ID	Term	Genes	Size	ExpCount	Count	Pvalue	Qvalue
1	GO:0005576	extracellular region	ADAMTSL2, BGN, CCDC80, CILP, CLEC11A, COL12A1, COL14A1, COL15A1, COL1A1, COL1A2, COL3A1, COL4A1, COL4A3, COL5A1, COL5A2, COL8A1, COL8A2, COMP, CRLF1, CTGF, CTHRC1, CTSK, DKK3, EDN3, EFEMP1, ELN, ENDOD1, ENPP1, FBLN1, FIBIN, FMOD, FN1, FRZB, GDF15, GPC6, ITGBL1, ITIH5, LAMA4, LAMC1, LGALS3, LOX, LOXL2, LOXL3, LTBP2, MFAP4, MFAP5, MMP2, NID1, NPPA, PAMR1, PDGFRL, POSTN, PRELP, PTN, SERPINE1, SERPINF1, SFRP2, SLC1A3, SPP1, SVEP1, TGFB2, THBS1, THBS4, TIMP1, TIMP2, TIMP3, TNC, WISP2	1559	12.35	68	3e-34	5e-31
2	GO:0005604	basement membrane	CCDC80, COL15A1, COL5A1, COL8A1, COL8A2, FBLN1, FN1, TGFB2, THBS4, TIMP1, TIMP2, TIMP3, TNC	57	0.44	13	3e-16	6e-13
3	GO:0005578	proteinaceous extracellular matrix	ADAMTSL2, BGN, CILP, COL14A1, COMP, CTGF, CTHRC1, EFEMP1, ELN, FMOD, GPC6, LGALS3, MFAP4, MMP2, POSTN, PRELP, PTN	164	1.08	17	6e-16	1e-12
4	GO:0072359	circulatory system development	COL1A1, COL1A2, COL3A1, COL4A1, COL4A3, COL5A1, COL8A1, COL8A2, CTGF, DDAH1, FN1, FRZB, FZD2, LAMA4, LOX, MMP2, MYH7, NOX4, PKD1, PTK2B, RTN4, RUNX1, SERPINE1, SERPINF1, SFRP2, TGFB2, THBS1, TNMD	546	4.28	28	2e-15	3e-12
5	GO:0001568	blood vessel development	COL1A1, COL1A2, COL3A1, COL4A1, COL4A3, COL5A1, COL8A1, COL8A2, CTGF, DDAH1, FN1, LAMA4, LOX, PKD1, PTK2B, RTN4, RUNX1, SERPINE1, SERPINF1, SFRP2, TGFB2, THBS1, TNMD	349	2.72	23	4e-15	6e-12
6	GO:0007275	multicellular organismal development	COL1A2, COL3A1, COL4A1, COL5A1, COL5A2, COL8A1, COL8A2, COMP, DKK3, EDN3, EGR3, ELN, ENPP1, FN1, IRS2, KDM5A, LAMC1, LGALS3, LRP1, MYH7, NFIC, NID1, NOX4, PDLIM7, PTK2B, PTN, RCAN1, SHISA3, SLC1A3, SPP1, SPRR1A, THBS4, TIMP1, TNC, UNC5B	1281	8.22	35	6e-14	1e-10
7	GO:0007155	cell adhesion	ACTN1, COL12A1, COL14A1, COL15A1, COL3A1, COL4A3, COL5A1, COL8A2, COMP, FN1, ITGBL1, LAMC1, MFAP4, PCDH17, POSTN, PTK2B, SVEP1, TNC, WISP2, ZYX	316	2.23	20	8e-14	1e-10
8	GO:0005615	extracellular space	CILP, COL12A1, COL14A1, COL15A1, COL1A1, COL1A2, COL3A1, COMP, CTGF, CTSK, DKK3, EDN3, EFEMP1, ENPP1, FBLN1, FMOD, FRZB, GDF15, LTBP2, MMP2, SERPINE1, SERPINF1, SFRP2, SPP1, TGFB2, THBS4, TIMP2, TNC	637	4.98	28	9e-14	2e-10
9	GO:0005201	extracellular matrix structural constituent	COL1A1, COL1A2, COL3A1, COL4A1, COL4A3, COL5A1, COL5A2, COMP, LAMC1	26	0.20	9	2e-13	4e-10
10	GO:0005581	collagen	COL1A1, COL1A2, COL3A1, COL4A1, COL4A3, COL5A1,	21	0.17	8	2e-12	4e-09

Table 2: Heart cluster 2. Genes in cluster: 2610507B11RIK, 4930534B04RIK, A530016L24RIK, ALDOB, ANGPT1, ANO10, AQP4, ATRX, BCL2L11, CENPF, CES3, CHCHD7, CLIP1, CTNNA1, ELF2, FBP2, GBF1, GPR22, HDAC11, KIF13A, KLF9, LGALS4, LUC7L2, NFIB, PARP1, PCP4, PFKFB1, PHKG1, PIM1, RET, RETNLA, SORBS1, TBC1D10C, TIRAP, TRDN, ZC3H11A, ZC3H13

	GO ID	Term	Genes	Size	ExpCount	Count	Pvalue	Qvalue
1	GO:0045787	positive regulation of cell cycle	BCL2L11, CENPF, PIM1	90	0.2	3	5e-04	0.4
2	GO:0005976	polysaccharide metabolic process	ANGPT1, PHKG1, SORBS1	117	0.2	3	1e-03	0.8
3	GO:0016051	carbohydrate biosynthetic process	ANGPT1, FBP2, PHKG1	121	0.2	3	1e-03	0.8
4	GO:0051347	positive regulation of transferase activity	ANGPT1, PFKFB1, PIM1, TIRAP	268	0.5	4	1e-03	0.9
