# Supplementary material for: Identifying pathogenic processes by integrating microarray data with prior knowledge
Source: BMC Bioinformatics. 2014 Apr 24;15:115. doi: 10.1186/1471-2105-15-115 (PMC4006456; doi:10.1186/1471-2105-15-115)
Supplement: Additional file 8 — GO results MCIP without priors, main cluster, heart failure data. Results of Gene ontology analysis of main heart failure cluster found using our method without priors. [file 1471-2105-15-115-S8.PDF]

Table 1: Genes in cluster: 1500009L16RIK, 2810474O19RIK, 4633401B06RIK, A530016L24RIK, AA414768, ACE, ACTA1, ACTN1, ADCY7, AHNAK2, ANGPTL7, ANKRD23, ASAP2, ASNS, ASPN, ATRX, BACE2, BBX, BCL2, BDH1, CAPG, CAR3, CCDC80, CDK1, CHD4, CHMP4C, CHN2, CHODL, CHPF, CILP, CKAP4, CLEC11A, CMKLR1, COL1A1, COL1A2, COL3A1, COL4A3, COL4A4, COL6A3, COL8A2, CRISPLD1, CRISPLD2, CRLF1, CTHRC1, CTSK, CUEDC1, CYGB, CYP1B1, DAB2IP, DBN1, DCLK1, E230013L22RIK, EFHD2, EFNB3, EIF2C2, ENAH, ENPP1, ETV5, FAM198B, FMOD, FN1, FOLR2, FRZB, FSTL3, FXYD6, FZD2, GPC6, HBEGF, HEATR6, HNMT, HR, IFT81, IGFBP6, IKBKAP, IMPA2, KIF13A, KIF26B, KRT18, LBP, LGI1, LMAN1L, LOX, LOXL2, MFAP5, MGP, MLKL, MLLT11, MMP14, MTAP1B, MYC, MYL7, NCEH1, NID1, NKTR, NLRC3, NOX4, NPPA, NUPR1, OLFML3, PAK1, PAMR1, PCDH9, PDE4DIP, PDGFRL, PDLIM5, PDZD3, PHLDA3, PLCB4, PLEKHH1, POSTN, PRELP, PTN, PTPRN, RAB27B, RASSF8, RCAN1, RDX, RFC1, RGS2, RHOU, RNF20, RUNX1, SEMA3F, SERPINE1, SFRP1, SFRP2, SHISA3, SLC12A5, SLC16A6, SLC1A3, SLC39A6, SLC41A2, SLTM, SMC4, SMC6, SNED1, SPNB2, SPP1, SPRR1A, STAR, SULF1, SVEP1, SYNPO2L, TCEAL7, TGFB3, THBS4, TRDN, TRIM59, TSPAN17, TTLL1, TTLL7, UCK2, UNC5B, USP36, ZBED6, ZC3H13

	GO ID	Term	Genes	Exp	Size	Count	Pval	Qval
1	GO:0005576	extracellular region	ACE, ACTN1, ANGPTL7, ASPN, CILP, CLEC11A, COL1A1, COL1A2, COL3A1, COL4A3, COL4A4, COL6A3, COL8A2, CRISPLD2, CRLF1, CTSK, ENPP1, FMOD, FN1, FOLR2, FRZB, FSTL3, HBEGF, IGFBP6, LBP, LGI1, LOX, LOXL2, MFAP5, MGP, NID1, NPPA, OLFML3, PAMR1, PDGFRL, POSTN, PRELP, PTN, SEMA3F, SERPINE1, SFRP1, SLC1A3, SNED1, SPP1, SULF1, SVEP1, TGFB3, THBS4	15.44	1450	48	7e-14	1e-10
2	GO:0005201	extracellular matrix structural constituent	COL1A1, COL1A2, COL3A1, COL4A3, COL4A4, COL8A2, FN1, MFAP5, MGP, PRELP	0.75	70	10	3e-09	6e-06
3	GO:0005615	extracellular space	ACE, CILP, COL1A1, COL1A2, COL3A1, COL6A3, CTSK, ENPP1, FMOD, FRZB, FSTL3, HBEGF, IGFBP6, LBP, LGI1, LOX, LOXL2, MGP, PTN, SEMA3F, SERPINE1, SFRP1, SPP1, SULF1, TGFB3, THBS4	7.17	684	26	7e-09	1e-05
4	GO:0005581	collagen	COL1A1, COL1A2, COL3A1, COL4A3, COL4A4, COL6A3, COL8A2, LOX	0.66	62	8	3e-07	5e-04
5	GO:0000902	cell morphogenesis	BCL2, CDK1, COL1A1, COL1A2, COL3A1, COL4A3, COL4A4, COL6A3, EFNB3, ENAH, FN1, FZD2, LGI1, LOXL2, NOX4, PAK1, PDLIM5, SEMA3F, SFRP1, SLC1A3, SPP1, TGFB3, THBS4, UNC5B	7.76	728	24	5e-07	1e-03
6	GO:0032836	glomerular basement membrane development	COL4A3, COL4A4, NID1, SULF1	0.10	9	4	1e-06	3e-03
7	GO:0042330	taxis	CDK1, CMKLR1, COL1A1, COL1A2, COL3A1, COL4A3, COL4A4, COL6A3, EFNB3, ENAH, LBP, LGI1, PAK1, SEMA3F, SERPINE1, SPP1, THBS4, UNC5B	5.46	512	18	7e-06	1e-02
