# Supplementary material for: Identifying pathogenic processes by integrating microarray data with prior knowledge
Source: BMC Bioinformatics. 2014 Apr 24;15:115. doi: 10.1186/1471-2105-15-115 (PMC4006456; doi:10.1186/1471-2105-15-115)
Supplement: Additional file 9 — GO results Kmeans clustering, main cluster, heart failure data. Results of Gene ontology analysis of main heart failure cluster found using Kmeans clustering. [file 1471-2105-15-115-S9.PDF]

Table 1: Genes in cluster: 0610040J01RIK, 1500009L16RIK, 1500015O10RIK, 2310010M20RIK, 2610507B11RIK, 3110040M04RIK, 4633401B06RIK, A530016L24RIK, ABCA4, ACBD3, ACE, ACTN1, ADAMTS2, AHNAK2, ANKRD23, ASAP2, ASNS, ASPN, ASS1, BAT2L2, BCL2, BTBD11, CAPG, CAR3, CES3, CHIC1, CHMP4C, CHODL, CKAP4, CLEC11A, CNKSR1, COL12A1, COL16A1, COL1A1, COL4A3, COL4A4, COL5A2, COL6A3, CPNE3, CPXM1, CREBBP, CRISPLD2, CTHRC1, CYGB, D7ERTD715E, DAB2IP, DCLK1, DIO2, DKK3, EFHD2, EFNB3, EGR3, ELF2, EMP1, FIBIN, FMOD, FRZB, FXYD6, FZD2, GDAP10, GDF15, GPC6, HBEGF, HNMT, HR, IGF1R, IKBKAP, ITGB1BP3, ITIH5, KCNJ14, KDM5A, KIF13A, KIF1B, KIF5B, KRT18, LBP, LEPREL1, LGALS3, LMAN1L, LOX, MFAP4, MFAP5, MGP, MICAL1, MID2, MLLT11, MTHFD2, MYL7, NID1, NKD2, NPPA, NPR3, OMD, OPN4, PAMR1, PANK3, PANX1, PDLIM2, PDPK1, PFKP, PHKG1, PLA2G5, PLEKHA4, PLEKHH1, PMEPA1, PTPRN, QSOX1, RASGRP3, RCAN1, RNF20, RSAD1, RUNX1, SCARF2, SCN4B, SERPINE1, SEZ6L2, SFRP1, SFRP2, SFRS2IP, SLC12A5, SLC16A7, SLC1A3, SLC22A3, SLC36A2, SLC41A2, SLITRK4, SLTM, SMC4, SMC6, SNED1, SPP1, SRPX2, STAR, SVEP1, SYNE1, TBX15, TCEAL7, TGFB2, TGFB3, TIMP1, TMEM45A, TNMD, TRIM47, TRIM59, TTLL1, TTLL7, TTN, USP7, VCAN, VGLL3, WISP2, ZBTB38, ZFP704

	GO ID	Term	Genes	Exp	Size	Count	Pval	Qval
1	GO:0005576	extracellular region	ACE, ACTN1, ADAMTS2, ASPN, CLEC11A, COL16A1, COL1A1, COL4A3, COL4A4, COL5A2, COL6A3, CRISPLD2, DKK3, FMOD, FRZB, GDF15, HBEGF, ITIH5, LBP, LEPREL1, LGALS3, LOX, MFAP4, MFAP5, MGP, NID1, NPPA, OMD, PAMR1, PLA2G5, QSOX1, SERPINE1, SFRP1, SLC1A3, SNED1, SPP1, SRPX2, SVEP1, TGFB2, TGFB3, TIMP1, TTN, VCAN, WISP2	14.30	1450	44	1e-12	2e-09
2	GO:0030199	collagen fibril organization	ADAMTS2, COL1A1, COL5A2, LOX, TGFB2	0.28	29	5	7e-06	1e-02
3	GO:0071842	cellular component organization at cellular level	ADAMTS2, COL1A1, COL4A3, COL4A4, COL5A2, COL6A3, CRISPLD2, EFNB3, FZD2, IGF1R, LGALS3, NID1, SPP1, TGFB2, TIMP1, VCAN	4.82	488	16	2e-05	3e-02
4	GO:0022601	menstrual cycle phase	SFRP1, TGFB2, TGFB3	0.07	7	3	3e-05	5e-02
5	GO:0005578	proteinaceous extracellular matrix	ADAMTS2, ASPN, FMOD, LGALS3, MGP, OMD, SFRP1, VCAN	1.28	145	8	4e-05	7e-02
6	GO:0005201	extracellular matrix structural constituent	COL1A1, COL4A3, COL4A4, COL5A2, MFAP5, MGP	0.67	70	6	5e-05	9e-02
7	GO:0032836	glomerular basement membrane development	COL4A3, COL4A4, NID1	0.09	9	3	7e-05	1e-01
8	GO:0031093	platelet alpha granule lumen	ACTN1, SERPINE1, TGFB2, TGFB3, TIMP1	0.45	46	5	8e-05	1e-01
9	GO:0043233	organelle lumen	ACTN1, SERPINE1, TGFB2, TGFB3, TIMP1	0.48	44	5	1e-04	2e-01
10	GO:0002576	platelet degranulation	ACTN1, SERPINE1, TGFB2, TGFB3, TIMP1, TTN	0.78	81	6	1e-04	2e-01
