# Supplementary material for: Identifying pathogenic processes by integrating microarray data with prior knowledge
Source: BMC Bioinformatics. 2014 Apr 24;15:115. doi: 10.1186/1471-2105-15-115 (PMC4006456; doi:10.1186/1471-2105-15-115)
Supplement: Additional file 11 — GO results MCIP with priors, melanoma data. Results of Gene ontology analysis of melanoma clusters found using our method with priors. [file 1471-2105-15-115-S11.PDF]

Table 1: Melanoma cluster 1. Genes in cluster: ABCA12, ALDH3A1, ALOX12B, ANXA9, APOC2, AQP3, ARG1, BBOX1, BIRC7, C1orf116, CALML3, CALML5, CD24, CDHR1, CDSN, CLCA2, CLCA4, CLDN1, CLIC3, CLTB, COL17A1, CR1, CRCT1, CST6, CSTA, CWH43, CXCL14, DEFB1, DEFB4A, DIO2, DSC1, DSC2, DSC3, DSG1, DSP, DST, DUOX1, ELOVL4, EPCAM, EPN3, EPS8L1, ESRP2, FERMT1, FGFBP1, FGFR2, FGFR3, FLG, GJB3, GJB5, GLTP, GPR87, GSTA3, HAL, HLF, HOXB7, HPGD, HSD11B2, IL1F7, IMPA2, IRX4, ITGB3, IVL, KLF5, KLK10, KLK11, KLK7, KRT1, KRT14, KRT15, KRT16, KRT17, KRT2, KRT23, KRT5, KRT6A, KRT6B, LAMA3, LCE2B, LGR5, LOR, LPHN3, LY6D, LYPD3, NAT, NEBL, PDZK1IP1, PI3, PKP1, PKP3, POU2F3, PPL, RAB25, RAPGEFL1, S100A14, S100A2, S100A7, SCEL, SCNN1A, SERPINB13, SERPINB3, SERPINB4, SERPINB5, SERPINB7, SFN, SLC16A4, SLURP1, SOX10, SPINK5, SPRR1A, SPRR1B, SRPX, TACSTD2, TFAP2B, TGM1, TGM3, TP63, TPSAB1, TRIM29, TUBB2B, UNC93A, VSNL1, ZNF750

	GO ID	Term	Genes	Exp	Size	Count	Pval	Qval
1	GO:0008544	epidermis development	ALOX12B, CALML5, COL17A1, CST6, CSTA, FLG, GJB5, IVL, KLK7, KRT1, KRT14, KRT15, KRT16, KRT17, KRT2, KRT5, LAMA3, LCE2B, LOR, POU2F3, PPL, S100A7, SCEL, SPRR1A, SPRR1B, TGM1	0.90	94	26	6e-32	8e-29
2	GO:0001533	cornified envelope	CDSN, CST6, CSTA, DSP, IVL, LOR, SCEL, SPRR1A, SPRR1B, TGM1	0.13	13	10	2e-18	3e-15
3	GO:0031424	keratinization	IVL, KRT2, LCE2B, LOR, PPL, SPRR1A, SPRR1B, TGM1, TGM3	0.17	16	9	1e-14	1e-11
4	GO:0030057	desmosome	CDSN, DSC1, DSC2, DSC3, DSG1, DSP, PKP1, PKP3, PPL	0.21	21	9	2e-13	3e-10
5	GO:0005200	structural constituent of cytoskeleton	DSP, KRT1, KRT14, KRT15, KRT16, KRT17, KRT2, KRT5, KRT6A, KRT6B, LOR, PPL	0.74	72	12	7e-12	9e-09
6	GO:0018149	peptide cross-linking	CSTA, DSP, IVL, LOR, SPRR1A, SPRR1B, TGM1, TGM3	0.25	24	8	7e-11	8e-08
7	GO:0030216	keratinocyte differentiation	CDSN, CSTA, DSP, FLG, POU2F3, S100A7, SCEL, SFN	0.29	31	8	3e-10	4e-07
8	GO:0030414	peptidase inhibitor activity	BIRC7, CST6, CSTA, PI3, SERPINB13, SERPINB3, SERPINB4, SERPINB5, SERPINB7, SPINK5, TFAP2B	1.22	119	11	3e-08	4e-05
9	GO:0061135	endopeptidase regulator activity	BIRC7, CST6, CSTA, PI3, SERPINB13, SERPINB3, SERPINB4, SERPINB5, SERPINB7, SPINK5, TFAP2B	1.23	120	11	4e-08	5e-05
10	GO:0031581	hemidesmosome assembly	COL17A1, DST, KRT14, KRT5, LAMA3	0.12	12	5	8e-08	1e-04

Table 2: Melanoma cluster 2. Genes in cluster: AIM1L, AKR1B10, ALDH3B2, DSG3, EGFR, EHF, ENDOU, EPHX3, GPR143, HLA-DQB2, HOPX, HSD17B2, JUP, KRT7, LAMC2, ME1, OVOL1, PRSS8, RHCG, RHOD, S100A1, SLC15A1, SOSTDC1, SPINT1, TNFRSF25, TP53AIP1, WNT4

	GO ID	Term	Genes	Exp	Size	Count	Pval	Qval
1	GO:0006702	androgen biosynthetic process	HSD17B2, WNT4	0.03	14	2	5e-04	0.4
2	GO:0005576	extracellular region	AKR1B10, EGFR, ENDOU, EPHX3, LAMC2, PRSS8, SOSTDC1, SPINT1, TNFRSF25, WNT4	3.16	1446	10	5e-04	0.4
3	GO:0030057	desmosome	DSG3, JUP	0.05	21	2	9e-04	0.6
4	GO:0048599	oocyte development	JUP, WNT4	0.05	22	2	1e-03	0.9
5	GO:0045168	cell-cell signaling involved in cell fate commitment	JUP, WNT4	0.07	28	2	2e-03	1.0
6	GO:0006081	cellular aldehyde metabolic process	AKR1B10, ALDH3B2	0.07	29	2	2e-03	1.0
7	GO:0002159	desmosome assembly	JUP	0.00	1	1	2e-03	1.0
8	GO:0006741	NADP biosynthetic process	ME1	0.00	1	1	2e-03	1.0
9	GO:0031945	positive regulation of glucocorticoid metabolic process	WNT4	0.00	1	1	2e-03	1.0
10	GO:0038030	non-canonical Wnt receptor signaling pathway via MAPK cascade	WNT4	0.00	1	1	2e-03	1.0

Table 3: Melanoma cluster 3. Genes in cluster: ALOXE3, ALX1, ANK3, C1orf46, CA2, CHP2, DUOX2, EDNRB, ENO2, HOOK1, INADL, KLF13, LY6G6C, MAGEA3, MYO6, POF1B, PTK6, QPRT, RBP1, SLC16A3, SOX15, TFPI2, WNT5A

	GO ID	Term	Genes	Exp	Size	Count	Pval	Qval
1	GO:0048853	forebrain morphogenesis	DUOX2, WNT5A	0.02	10	2	1e-04	0.08
2	GO:0033189	response to vitamin A	PTK6, RBP1, WNT5A	0.15	91	3	4e-04	0.32
3	GO:0014043	negative regulation of neuron maturation	EDNRB	0.00	1	1	2e-03	1.00
4	GO:0034213	quinolinate catabolic process	QPRT	0.00	1	1	2e-03	1.00
5	GO:0035645	enteric smooth muscle cell differentiation	EDNRB	0.00	1	1	2e-03	1.00
6	GO:0048855	adenohypophysis morphogenesis	DUOX2	0.00	1	1	2e-03	1.00
7	GO:0060210	metestrus	WNT5A	0.00	1	1	2e-03	1.00
8	GO:0071300	cellular response to retinoic acid	PTK6, WNT5A	0.07	40	2	2e-03	1.00
9	GO:0010976	positive regulation of neuron projection development	PTK6, WNT5A	0.07	42	2	2e-03	1.00
10	GO:0021536	diencephalon development	DUOX2, WNT5A	0.08	49	2	3e-03	1.00

Table 4: Melanoma cluster 4. Genes in cluster: ACPP, AKR1C2, CEACAM6, CYP2E1, CYP39A1, EFS, EPB41L4B, EVPL, F2RL1, GRHL2, HR, KLK5, KRT34, KRT75, LTF, MIA, PDZD2, PERP, PHACTR1, PLCB4, PRAME, SLC22A3, SOX9, SPINT2, SPRR3

	GO ID	Term	Genes	Exp	Size	Count	Pval	Qval
1	GO:0031424	keratinization	EVPL, SPRR3	0.03	16	2	4e-04	0.2
2	GO:0018149	peptide cross-linking	EVPL, SPRR3	0.04	24	2	9e-04	0.5
3	GO:0030057	desmosome	EVPL, PERP	0.05	21	2	9e-04	0.6
4	GO:0003415	chondrocyte hypertrophy	SOX9	0.00	1	1	2e-03	1.0
5	GO:0034137	positive regulation of toll-like receptor 2 signaling pathway	F2RL1	0.00	1	1	2e-03	1.0
6	GO:0035622	intrahepatic bile duct development	SOX9	0.00	1	1	2e-03	1.0
7	GO:0035926	chemokine (C-C motif) ligand 2 secretion	F2RL1	0.00	1	1	2e-03	1.0
8	GO:0043311	positive regulation of eosinophil degranulation	F2RL1	0.00	1	1	2e-03	1.0
9	GO:0051615	histamine uptake	SLC22A3	0.00	1	1	2e-03	1.0
10	GO:0060221	retinal rod cell differentiation	SOX9	0.00	1	1	2e-03	1.0

Table 5: Melanoma cluster 5. Genes in cluster: C10orf116, CBLC, CYP2C18, FZD10, LASS4, MARCO, PEG10

	GO ID	Term	Genes	Exp	Size	Count	Pval	Qval
1	GO:0008390	testosterone 16-alpha-hydroxylase activity	CYP2C18	0.00	1	1	6e-04	0.1
2	GO:0034259	negative regulation of Rho GTPase activity	FZD10	0.00	3	1	2e-03	0.3
3	GO:0007175	negative regulation of epidermal growth factor-activated receptor activity	CBLC	0.00	6	1	3e-03	0.6
4	GO:0034260	negative regulation of GTPase activity	FZD10	0.00	8	1	4e-03	0.8
5	GO:0032855	positive regulation of Rac GTPase activity	FZD10	0.01	20	1	1e-02	1.0
6	GO:0035567	non-canonical Wnt receptor signaling pathway	FZD10	0.01	24	1	1e-02	1.0
7	GO:0030512	negative regulation of transforming growth factor beta receptor signaling pathway	PEG10	0.02	34	1	2e-02	1.0
8	GO:0061097	regulation of protein tyrosine kinase activity	CBLC	0.02	34	1	2e-02	1.0
9	GO:0071300	cellular response to retinoic acid	FZD10	0.02	40	1	2e-02	1.0
10	GO:0009968	negative regulation of signal transduction	CBLC, PEG10	0.25	447	2	2e-02	1.0

Table 6: Melanoma cluster 6. Genes in cluster: ALOX12, CDS1, GDPD3, HOXD13, KCNJ12, KLK6, MAGEA6, PLA1A, SLPI, SULT2B1, TPSB2

	GO ID	Term	Genes	Exp	Size	Count	Pval	Qval
1	GO:0004027	alcohol sulfotransferase activity	SULT2B1	0.00	1	1	0.001	0.3
2	GO:0016024	CDP-diacylglycerol biosynthetic process	CDS1	0.00	1	1	0.001	0.3
3	GO:0048619	embryonic hindgut morphogenesis	HOXD13	0.00	1	1	0.001	0.3
4	GO:0004605	phosphatidate cytidylyltransferase activity	CDS1	0.00	2	1	0.002	0.6
5	GO:0008970	phosphatidylcholine 1-acylhydrolase activity	PLA1A	0.00	2	1	0.002	0.6
6	GO:0047977	hepoxilin-epoxide hydrolase activity	ALOX12	0.00	2	1	0.002	0.6
7	GO:0004052	arachidonate 12-lipoxygenase activity	ALOX12	0.00	3	1	0.003	0.8
8	GO:0004142	diacylglycerol cholinephosphotransferase activity	CDS1	0.00	3	1	0.003	0.8
9	GO:0050294	steroid sulfotransferase activity	SULT2B1	0.00	3	1	0.003	0.8
10	GO:0000103	sulfate assimilation	SULT2B1	0.00	3	1	0.003	0.8

Table 7: Melanoma cluster 7. Genes in cluster: ABLIM1, CYP4F12, FCER1A, GATA3, IL1F9, KCND3, KRT19, LAD1, SCNN1B, TPTE, TUBA4A, VIPR1

	GO ID	Term	Genes	Exp	Size	Count	Pval	Qval
1	GO:0061217	regulation of mesonephros development	GATA3	0.00	1	1	9e-04	0.4
2	GO:2000607	negative regulation of cell proliferation involved in mesonephros development	GATA3	0.00	1	1	9e-04	0.4
3	GO:2000703	negative regulation of fibroblast growth factor receptor signaling pathway involved in ureteric bud formation	GATA3	0.00	1	1	9e-04	0.4
4	GO:2000734	negative regulation of glial cell-derived neurotrophic factor receptor signaling pathway involved in ureteric bud formation	GATA3	0.00	1	1	9e-04	0.4
5	GO:0001812	positive regulation of type I hypersensitivity	FCER1A	0.00	2	1	2e-03	0.9
6	GO:0032672	regulation of interleukin-3 production	FCER1A	0.00	2	1	2e-03	0.9
7	GO:0045401	positive regulation of interleukin-3 biosynthetic process	FCER1A	0.00	2	1	2e-03	0.9
8	GO:0060995	cell-cell signaling involved in kidney development	GATA3	0.00	2	1	2e-03	0.9
9	GO:0061290	canonical Wnt receptor signaling pathway involved in metanephric kidney development	GATA3	0.00	2	1	2e-03	0.9
10	GO:2000664	positive regulation of interleukin-5 secretion	GATA3	0.00	2	1	2e-03	0.9

Table 8: Melanoma cluster 8. Genes in cluster: C1orf106, CA8, CLDN4, EMILIN1, FXYD3, GLUD2, KLK8, MPZL2, SPRR2C

	GO ID	Term	Genes	Exp	Size	Count	Pval	Qval
1	GO:0004352	glutamate dehydrogenase (NAD+) activity	GLUD2	0.00	2	1	0.001	0.2
2	GO:0004353	glutamate dehydrogenase [NAD(P)+] activity	GLUD2	0.00	2	1	0.001	0.2
3	GO:0031642	negative regulation of myelination	KLK8	0.00	3	1	0.002	0.3
4	GO:0030023	extracellular matrix constituent conferring elasticity	EMILIN1	0.00	3	1	0.002	0.3
5	GO:0070728	leucine binding	GLUD2	0.00	3	1	0.002	0.3
6	GO:0048681	negative regulation of axon regeneration	KLK8	0.00	5	1	0.003	0.5
7	GO:0006537	glutamate biosynthetic process	GLUD2	0.00	6	1	0.004	0.6
8	GO:0006538	glutamate catabolic process	GLUD2	0.00	7	1	0.005	0.7
9	GO:0070570	regulation of neuron projection regeneration	KLK8	0.01	11	1	0.007	1.0
10	GO:0016338	calcium-independent cell-cell adhesion	CLDN4	0.01	16	1	0.010	1.0

Table 9: Melanoma cluster 9. Genes in cluster: ANXA3, AP1M2, C1orf68, CDH3, GREB1, IL20RA, KLK13, LAMB3, PLAT, PRSS2, PRSS3

	GO ID	Term	Genes	Exp	Size	Count	Pval	Qval
1	GO:0004252	serine-type endopeptidase activity	KLK13, PLAT, PRSS2, PRSS3	0.09	110	4	1e-06	2e-04
2	GO:0017171	serine hydrolase activity	KLK13, PLAT, PRSS2, PRSS3	0.11	127	4	2e-06	4e-04
3	GO:0070011	peptidase activity, acting on L-amino acid peptides	KLK13, PLAT, PRSS2, PRSS3	0.34	406	4	2e-04	4e-02
4	GO:0005509	calcium ion binding	ANXA3, CDH3, PRSS2, PRSS3	0.43	513	4	5e-04	1e-01
5	GO:0005610	laminin-5 complex	LAMB3	0.00	2	1	2e-03	3e-01
6	GO:0019834	phospholipase A2 inhibitor activity	ANXA3	0.00	3	1	2e-03	4e-01
7	GO:0007586	digestion	PRSS2, PRSS3	0.08	93	2	3e-03	5e-01
8	GO:0005615	extracellular space	KLK13, PLAT, PRSS2, PRSS3	0.66	683	4	3e-03	5e-01
9	GO:0031638	zymogen activation	PRSS3	0.00	5	1	4e-03	7e-01
10	GO:0034329	cell junction assembly	CDH3, LAMB3	0.12	142	2	6e-03	1e+00

Table 10: Melanoma cluster 10. Genes in cluster: AKR1C3, CA12, CDH1, CDH13, IL1RN, LSR, RAB27B, SLC6A14, TMPRSS11D, TNFSF10, TTC9, VSIG4

	GO ID	Term	Genes	Exp	Size	Count	Pval	Qval
1	GO:0034332	adherens junction organization	CDH1, CDH13	0.04	36	2	6e-04	0.2
2	GO:0005150	interleukin-1, Type I receptor binding	IL1RN	0.00	1	1	1e-03	0.5
3	GO:0035410	dihydrotestosterone 17-beta-dehydrogenase activity	AKR1C3	0.00	1	1	1e-03	0.5
4	GO:0045352	interleukin-1 Type I receptor antagonist activity	IL1RN	0.00	1	1	1e-03	0.5
5	GO:0045353	interleukin-1 Type II receptor antagonist activity	IL1RN	0.00	1	1	1e-03	0.5
6	GO:0045703	ketoreductase activity	AKR1C3	0.00	1	1	1e-03	0.5
7	GO:0047017	prostaglandin-F synthase activity	AKR1C3	0.00	1	1	1e-03	0.5
8	GO:0047020	15-hydroxyprostaglandin-D dehydrogenase (NADP+) activity	AKR1C3	0.00	1	1	1e-03	0.5
9	GO:0055100	adiponectin binding	CDH13	0.00	1	1	1e-03	0.5
10	GO:0002138	retinoic acid biosynthetic process	AKR1C3	0.00	2	1	2e-03	0.8

Table 11: Melanoma cluster 11. Genes in cluster: ATP2C2, CYP3A5, EPS8L2, GDF15, KRT10, MAGEA12, MALL, MS4A4A, NPR3, RENBP, RORA, SUSD4

	GO ID	Term	Genes	Exp	Size	Count	Pval	Qval
1	GO:0050121	N-acylglucosamine 2-epimerase activity	RENBP	0.00	1	1	9e-04	0.2
2	GO:0002158	osteoclast proliferation	NPR3	0.00	2	1	2e-03	0.4
3	GO:0009822	alkaloid catabolic process	CYP3A5	0.00	3	1	3e-03	0.5
4	GO:0004476	mannose-6-phosphate isomerase activity	RENBP	0.00	3	1	3e-03	0.6
5	GO:0016941	natriuretic peptide receptor activity	NPR3	0.00	3	1	3e-03	0.6
6	GO:0008217	regulation of blood pressure	NPR3, RENBP	0.10	117	2	4e-03	0.8
7	GO:0070989	oxidative demethylation	CYP3A5	0.01	8	1	7e-03	1.0
8	GO:0021692	cerebellar Purkinje cell layer morphogenesis	RORA	0.01	10	1	8e-03	1.0
9	GO:0021702	cerebellar Purkinje cell differentiation	RORA	0.01	10	1	8e-03	1.0
10	GO:0035810	positive regulation of urine volume	NPR3	0.01	10	1	8e-03	1.0

Table 12: Melanoma cluster 12. Genes in cluster: BNC1, COL7A1, CRISP3, EDN1, EXPH5, HOXB6, KRT33A, MAP7, MBP, S100A9, SLC6A15, TBX1, TMEM45A

	GO ID	Term	Genes	Exp	Size	Count	Pval	Qval
1	GO:0042474	middle ear morphogenesis	EDN1, TBX1	0.01	13	2	7e-05	0.03
2	GO:0021644	vagus nerve morphogenesis	TBX1	0.00	1	1	1e-03	0.47
3	GO:0031583	activation of phospholipase D activity by G-protein coupled receptor protein signaling pathway	EDN1	0.00	1	1	1e-03	0.47
4	GO:0042313	protein kinase C deactivation	EDN1	0.00	1	1	1e-03	0.47
5	GO:0005298	proline:sodium symporter activity	SLC6A15	0.00	1	1	1e-03	0.51
6	GO:0031707	endothelin A receptor binding	EDN1	0.00	1	1	1e-03	0.51
7	GO:0005590	collagen type VII	COL7A1	0.00	1	1	1e-03	0.52
8	GO:0048704	embryonic skeletal system morphogenesis	HOXB6, TBX1	0.06	57	2	1e-03	0.68
9	GO:0015820	leucine transport	SLC6A15	0.00	2	1	2e-03	0.93
10	GO:0035524	proline transmembrane transport	SLC6A15	0.00	2	1	2e-03	0.93

Table 13: Melanoma cluster 13. Genes in cluster: CD163, CD300A, KLF4, MAST4, NMU, PKP2, PSORS1C2, SERPINE2, SPP1, SPTLC3, TMPRSS4

	GO ID	Term	Genes	Exp	Size	Count	Pval	Qval
1	GO:0005044	scavenger receptor activity	CD163, TMPRSS4	0.03	31	2	4e-04	0.1
2	GO:0048685	negative regulation of collateral sprouting of intact axon in response to injury	SPP1	0.00	1	1	9e-04	0.3
3	GO:0030155	regulation of cell adhesion	KLF4, SERPINE2, SPP1	0.21	221	3	9e-04	0.3
4	GO:0001010	sequence-specific DNA binding transcription factor recruiting transcription factor activity	KLF4	0.00	1	1	1e-03	0.3
5	GO:0042922	neuromedin U receptor binding	NMU	0.00	1	1	1e-03	0.3
6	GO:0007500	mesodermal cell fate determination	KLF4	0.00	2	1	2e-03	0.6
7	GO:0014740	negative regulation of muscle hyperplasia	KLF4	0.00	2	1	2e-03	0.6
8	GO:0046985	positive regulation of hemoglobin biosynthetic process	KLF4	0.00	2	1	2e-03	0.6
9	GO:2000342	negative regulation of chemokine (C-X-C motif) ligand 2 production	KLF4	0.00	2	1	2e-03	0.6
10	GO:0090051	negative regulation of cell migration involved in sprouting angiogenesis	KLF4	0.00	3	1	3e-03	0.9

Table 14: Melanoma cluster 14. Genes in cluster: ASS1, BAMBI, BCL11A, BCL11B, BCL2A1, CITED1, CRNN, EFNA3, FAT2, FKBP10, GJB1, HSPC159, MAGEA1, METRN, PHLDA1, SDC1, SERPINB2, SLC6A2

	GO ID	Term	Genes	Exp	Size	Count	Pval	Qval
1	GO:0010720	positive regulation of cell development	BAMBI, BCL11A, METRN	0.20	126	3	0.001	0.4
2	GO:0010769	regulation of cell morphogenesis involved in differentiation	BAMBI, BCL11A, METRN	0.22	135	3	0.001	0.5
3	GO:0004055	argininosuccinate synthase activity	ASS1	0.00	1	1	0.001	0.7
4	GO:0005334	norepinephrine:sodium symporter activity	SLC6A2	0.00	1	1	0.001	0.7
5	GO:0021773	striatal medium spiny neuron differentiation	BCL11B	0.00	1	1	0.002	0.7
6	GO:0071105	response to interleukin-11	CITED1	0.00	1	1	0.002	0.7
7	GO:0051591	response to cAMP	CITED1, SDC1	0.07	46	2	0.002	0.9
8	GO:0000053	argininosuccinate metabolic process	ASS1	0.00	2	1	0.003	1.0
9	GO:0010046	response to mycotoxin	ASS1	0.00	2	1	0.003	1.0
10	GO:0045210	FasL biosynthetic process	PHLDA1	0.00	2	1	0.003	1.0

Table 15: Melanoma cluster 15. Genes in cluster: AHNAK2, ARHGEF4, CDA, DUSP4, FOXE1, GPR109B, GPX2, IRF6, MSMB, MYCN, PAEP, PAK6, PPP1R13L

	GO ID	Term	Genes	Exp	Size	Count	Pval	Qval
1	GO:0060465	pharynx development	FOXE1	0.00	1	1	0.001	0.3
2	GO:0071217	cellular response to external biotic stimulus	CDA	0.00	1	1	0.001	0.3
3	GO:0005634	nucleus	AHNAK2, DUSP4, FOXE1, GPX2, IRF6, MSMB, MYCN, PAEP, PAK6, PPP1R13L	4.54	4320	10	0.002	0.5
4	GO:0042633	hair cycle	FOXE1, PPP1R13L	0.07	64	2	0.002	0.5
5	GO:0019858	cytosine metabolic process	CDA	0.00	2	1	0.002	0.6
6	GO:0046898	response to cycloheximide	CDA	0.00	3	1	0.003	0.8
7	GO:0060022	hard palate development	FOXE1	0.01	5	1	0.005	1.0
8	GO:0060023	soft palate development	FOXE1	0.01	5	1	0.005	1.0
9	GO:0009972	cytidine deamination	CDA	0.01	7	1	0.007	1.0
10	GO:0043097	pyrimidine nucleoside salvage	CDA	0.01	7	1	0.007	1.0

Table 16: Melanoma cluster 16. Genes in cluster: AGR2, CDH22, HYAL4, MMP28, PLA2R1, SH2D3A, SLC24A3, STYK1, TGM2, TREX2

	GO ID	Term	Genes	Exp	Size	Count	Pval	Qval
1	GO:0002163	alpha-dystroglycan binding	AGR2	0.00	1	1	9e-04	0.2
2	GO:0018153	isopeptide cross-linking via N6-(L-isoglutamyl)-L-lysine	TGM2	0.00	2	1	2e-03	0.3
3	GO:0090238	positive regulation of arachidonic acid secretion	PLA2R1	0.00	2	1	2e-03	0.3
4	GO:0008273	calcium, potassium:sodium antiporter activity	SLC24A3	0.00	2	1	2e-03	0.4
5	GO:0090403	oxidative stress-induced premature senescence	PLA2R1	0.00	3	1	3e-03	0.5
6	GO:0060662	salivary gland cavitation	TGM2	0.00	4	1	3e-03	0.7
7	GO:0070254	mucus secretion	AGR2	0.00	4	1	3e-03	0.7
8	GO:0008296	3'-5'-exodeoxyribonuclease activity	TREX2	0.00	4	1	4e-03	0.7
9	GO:0008853	exodeoxyribonuclease III activity	TREX2	0.00	4	1	4e-03	0.7
10	GO:0007342	fusion of sperm to egg plasma membrane	HYAL4	0.01	7	1	6e-03	1.0

Table 17: Melanoma cluster 17. Genes in cluster: EPHB6, GAGE3, GJA1, IL1R2, LOC51152, NDRG4, PITX1, PTGER3, SFTPD, SMPD3

	GO ID	Term	Genes	Exp	Size	Count	Pval	Qval
1	GO:0010232	vascular transport	GJA1	0.00	1	1	7e-04	0.2
2	GO:0004910	interleukin-1, Type II, blocking receptor activity	IL1R2	0.00	2	1	1e-03	0.3
3	GO:0000137	Golgi cis cisterna	SMPD3	0.00	3	1	2e-03	0.5
4	GO:0014707	branchiomeric skeletal muscle development	PITX1	0.00	3	1	2e-03	0.6
5	GO:0015867	ATP transport	GJA1	0.00	3	1	2e-03	0.6
6	GO:0045085	negative regulation of interleukin-2 biosynthetic process	SFTPD	0.00	3	1	2e-03	0.6
7	GO:0015865	purine nucleotide transport	GJA1	0.00	4	1	3e-03	0.8
8	GO:0016264	gap junction assembly	GJA1	0.00	4	1	3e-03	0.8
9	GO:0004767	sphingomyelin phosphodiesterase activity	SMPD3	0.00	5	1	3e-03	0.8
10	GO:0004957	prostaglandin E receptor activity	PTGER3	0.00	5	1	3e-03	0.8

Table 18: Melanoma cluster 18. Genes in cluster: AKR1C1, ARL14, IL22RA1, NLRP1, PLEKHB1, RNFT2

	GO ID	Term	Genes	Exp	Size	Count	Pval	Qval
1	GO:0072558	NLRP1 inflammasome complex	NLRP1	0.00	1	1	5e-04	0.07
2	GO:0047006	17-alpha,20-alpha-dihydroxypregn-4-en-3-one dehydrogenase activity	AKR1C1	0.00	2	1	1e-03	0.16
3	GO:0047042	androsterone dehydrogenase (B-specific) activity	AKR1C1	0.00	2	1	1e-03	0.16
4	GO:0018636	phenanthrene 9,10-monooxygenase activity	AKR1C1	0.00	3	1	2e-03	0.23
5	GO:0047086	ketosteroid monooxygenase activity	AKR1C1	0.00	3	1	2e-03	0.23
6	GO:0047115	trans-1,2-dihydrobenzene-1,2-diol dehydrogenase activity	AKR1C1	0.00	3	1	2e-03	0.23
7	GO:0047718	indanol dehydrogenase activity	AKR1C1	0.00	3	1	2e-03	0.23
8	GO:0046683	response to organophosphorus	AKR1C1	0.00	4	1	2e-03	0.25
9	GO:0071395	cellular response to jasmonic acid stimulus	AKR1C1	0.00	4	1	2e-03	0.25
10	GO:0004904	interferon receptor activity	IL22RA1	0.00	5	1	3e-03	0.37
