# Supplementary material for: Identifying pathogenic processes by integrating microarray data with prior knowledge
Source: BMC Bioinformatics. 2014 Apr 24;15:115. doi: 10.1186/1471-2105-15-115 (PMC4006456; doi:10.1186/1471-2105-15-115)
Supplement: Additional file 12 — GO results MCIP without priors, main cluster, melanoma cancer data. Results of Gene ontology analysis of main melanoma cluster found using our method without priors. [file 1471-2105-15-115-S12.PDF]

Table 1: Genes in cluster: AKR1C1, ALOX12B, ARG1, C10orf116, C1orf116, CALML5, CDSN, CST6, DACH1, DEFB1, DSC1, ENDOU, ESRP2, GJB5, GPX2, HAL, HOPX, IL1R2, KLF4, KLK10, KLK5, KLK7, KRT10, KRT14, KRT2, LY6G6C, MALL, MIA, NMU, PERP, PITX1, PKP1, PRSS3, PTPRF, SERPINB7, SLC6A14, SLPI, SLURP1, SMPD3, ZNF185, ZNF750

| | GO ID | Term | Genes | Exp | Size | Count | Pval | Qval |
|---|---|---|---|---|---|---|---|---|
| 1 | GO:0008544 | epidermis development | ALOX12B, CALML5, CDSN, CST6, GJB5, KLK5, KLK7, KRT10, KRT14, KRT2, ZNF750 | 0.72 | 202 | 11 | 6e-11 | 4e-08 |
| 2 | GO:0030057 | desmosome | CDSN, DSC1, PERP, PKP1 | 0.07 | 21 | 4 | 7e-07 | 4e-04 |
| 3 | GO:0097209 | epidermal lamellar body | KLK5, KLK7 | 0.01 | 3 | 2 | 3e-05 | 2e-02 |
| 4 | GO:0045095 | keratin filament | KRT10, KRT14, KRT2 | 0.09 | 25 | 3 | 8e-05 | 4e-02 |
| 5 | GO:0005576 | extracellular region | ARG1, CDSN, CST6, DEFB1, ENDOU, IL1R2, KLK10, KLK5, KLK7, MIA, NMU, PRSS3, SLPI, SLURP1 | 4.94 | 1450 | 14 | 2e-04 | 1e-01 |
| 6 | GO:0030280 | structural constituent of epidermis | KRT10, PKP1 | 0.02 | 7 | 2 | 2e-04 | 1e-01 |
| 7 | GO:0004252 | serine-type endopeptidase activity | KLK10, KLK5, KLK7, PRSS3 | 0.38 | 112 | 4 | 5e-04 | 3e-01 |
| 8 | GO:0001533 | cornified envelope | CDSN, CST6 | 0.05 | 14 | 2 | 1e-03 | 5e-01 |
| 9 | GO:0001010 | sequence-specific DNA binding transcription factor recruiting transcription factor activity | KLF4 | 0.00 | 1 | 1 | 3e-03 | 1e+00 |
| 10 | GO:0004397 | histidine ammonia-lyase activity | HAL | 0.00 | 1 | 1 | 3e-03 | 1e+00 |
