# Supplementary material for: Identifying pathogenic processes by integrating microarray data with prior knowledge
Source: BMC Bioinformatics. 2014 Apr 24;15:115. doi: 10.1186/1471-2105-15-115 (PMC4006456; doi:10.1186/1471-2105-15-115)
Supplement: Additional file 13 — GO results Kmeans clustering, main cluster, melanoma cancer data. Results of Gene ontology analysis of main melanoma cluster found using Kmeans clustering. [file 1471-2105-15-115-S13.PDF]

Table 1: Genes in cluster: ANXA9, ARG1, BNC1, C1orf116, C1orf46, CALML3, CDSN, CWH43, EDN1, ENDOU, EVPL, FGFBP1, GPX2, HOPX, HOXB6, ITGB3, IVL, KCND3, KLK10, KLK11, KLK5, KLK7, KLK8, KRT10, KRT2, LAMB3, LY6G6C, PITX1, PRSS2, PRSS3, PSORS1C2, RAPGEFL1, SERPINB13, SLC6A2, SLURP1, SMPD3, SPP1, SPRR3, TGM1, TMPRSS11D, VIPR1

	GO ID	Term	Genes	Exp	Size	Count	Pval	Qval
1	GO:0008544	epidermis development	BNC1, CDSN, EVPL, IVL, KLK5, KLK7, KRT10, KRT2, LAMB3, SPRR3, TGM1	0.70	202	11	5e-11	3e-08
2	GO:0031424	keratinization	EVPL, IVL, KRT2, SPRR3, TGM1	0.05	15	5	1e-09	7e-07
3	GO:0004252	serine-type endopeptidase activity	KLK10, KLK11, KLK5, KLK7, KLK8, PRSS2, PRSS3, TMPRSS11D	0.39	112	8	3e-09	2e-06
4	GO:0001533	cornified envelope	CDSN, EVPL, IVL, TGM1	0.05	14	4	1e-07	7e-05
5	GO:0005576	extracellular region	ARG1, CDSN, EDN1, ENDOU, FGFBP1, KLK10, KLK11, KLK5, KLK7, KLK8, LAMB3, PRSS2, PRSS3, PSORS1C2, SERPINB13, SLURP1, SPP1, TMPRSS11D	4.94	1450	18	3e-07	2e-04
6	GO:0018149	peptide cross-linking	EVPL, IVL, SPRR3, TGM1	0.08	24	4	1e-06	8e-04
7	GO:0060429	epithelium development	CDSN, EVPL, IVL, KRT10, KRT2, SPRR3, TGM1	0.62	180	7	2e-06	1e-03
8	GO:0048731	system development	ARG1, BNC1, CDSN, EVPL, HOXB6, IVL, KLK5, KLK7, KRT10, KRT2, LAMB3, SPRR3, TGM1	3.23	933	13	5e-06	4e-03
9	GO:0008233	peptidase activity	ENDOU, KLK10, KLK11, KLK5, KLK7, KLK8, PRSS2, PRSS3, TMPRSS11D	1.46	422	9	1e-05	6e-03
10	GO:0097209	epidermal lamellar body	KLK5, KLK7	0.01	3	2	3e-05	2e-02
